# Cadherin‐11 increases tumor cell proliferation and metastatic potential via Wnt pathway activation

**DOI:** 10.1002/1878-0261.13507

**Published:** 2023-09-08

**Authors:** Yayu Liu, Pedro Lei, Ronel Z. Samuel, Anagha M. Kashyap, Theodore Groth, Wiam Bshara, Sriram Neelamegham, Stelios T. Andreadis

**Affiliations:** ^1^ Department of Chemical and Biological Engineering, University at Buffalo The State University of New York Amherst NY USA; ^2^ Roswell Park Comprehensive Cancer Center Pathology Resource Network Buffalo NY USA; ^3^ Department of Biomedical Engineering, University at Buffalo The State University of New York Amherst NY USA; ^4^ New York State Center of Excellence in Bioinformatics and Life Sciences Buffalo NY USA; ^5^ Center for Cell, Gene and Tissue Engineering (CGTE), University at Buffalo The State University of New York Amherst NY USA

**Keywords:** breast cancer, cadherin‐11, metastasis, Wnt signaling, β‐catenin

## Abstract

During epithelial–mesenchymal transition (EMT) in cancer progression, tumor cells switch cadherin profile from E‐cadherin to cadherin‐11 (CDH11), which is accompanied by increased invasiveness and metastatic activity. However, the mechanism through which CDH11 may affect tumor growth and metastasis remains elusive. Here, we report that CDH11 was highly expressed in multiple human tumors and was localized on the membrane, in the cytoplasm and, surprisingly, also in the nucleus. Interestingly, β‐catenin remained bound to carboxy‐terminal fragments (CTFs) of CDH11, the products of CDH11 cleavage, and co‐localized with CTFs in the nucleus in the majority of breast cancer samples. Binding of β‐catenin to CTFs preserved β‐catenin activity, whereas inhibiting CDH11 cleavage led to β‐catenin phosphorylation and diminished Wnt signaling, similar to *CDH11* knockout. Our data elucidate a previously unknown role of CDH11, which serves to stabilize β‐catenin in the cytoplasm and facilitates its translocation to the nucleus, resulting in activation of Wnt signaling, with subsequent increased proliferation, migration and invasion potential.

## Graphical abstract

This study elucidated the mechanism of Wnt activation by cadherin‐11 (CDH11). The intracellular domain of CDH11 binds to and stabilizes β‐catenin. In addition to localization in the membrane and cytoplasm, these proteins co‐localize in the nucleus, activating the Wnt/β‐catenin signaling pathway and ultimately enhancing proliferation, migration and invasiveness of breast cancer cells.

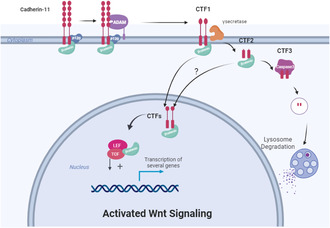

**Abbreviation**
ADAMs: a disintegrin and metalloendoproteinase
CDH1: cadherin‐1/E‐cadherin
CDH11: cadherin‐11/OB‐cadherin
CDH2: cadherin‐2/N‐cadherin
CTFs: carboxy‐terminal fragments
DF: dermal fibroblast
DMEM: Dulbecco's Modified Eagle medium
DTT: dithiothreitol
EC: extracellular domain
ECM: extracellular matrix
EDTA: ethylenediaminetetraacetic acid
EMT: epithelial–mesenchymal transition
ER: estrogen receptor
FBS: fetal bovine serum
FL: full length
FZD2/7: Frizzled2/7
GEPIA: gene expression profiling interactive analysis
HER2: human epidermal‐growth‐factor receptor 2
HF‐MSC: hair follicle‐derived mesenchymal stem cell
HRP: horseradish peroxidase
ID: intracellular domain
IP: immuno‐precipitation
KO: knock out
LRP: lipoprotein receptor‐related protein
M.S.: median score
MMP: metalloproteinase
MST1/2: mammalian Sterile20‐like kinase 1/2
PAR: paraffin archive resource
PR: progesterone receptor
PVDF: polyvinylidene fluoride
ROCK: Rho‐associated protein kinase
RPCCC: Roswell Park Comprehensive Cancer Center
TCF: T cell factor
TCGA: The Cancer Genome Atlas
TMA: tissue microarray
TNBC: triple‐negative breast cancer
YAP: yes‐associated protein

## 1 Introduction

Triple‐negative breast cancer (TNBC) is characterized by deficiency in estrogen receptor (ER), progesterone receptor (PR) and human epidermal growth factor receptor 2 (HER2) expression and represents one of the most aggressive subtypes of breast cancer [1]. TNBC is associated with poor prognosis, early aggressive metastasis, early recurrence, and a lower survival rate as well as limited chemotherapeutic options when compared with other subtypes of breast carcinoma [2, 3, 4].

The Wnt/β‐catenin signaling has been shown to be dysregulated in TNBC and plays a critical role in the initiation of breast tumorigenesis, progression, metastasis as well as development of chemotherapy resistance [5, 6]. Canonical Wnt/β‐catenin signaling is induced by Wnt ligands, and subsequently, stabilized β‐catenin accumulates in the cytoplasm followed by translocation into the nucleus and triggers transcription of Wnt target genes [7]. Aberrant accumulation of nuclear β‐catenin is more prevalent in TNBC when compared with other breast cancer subtypes and associated with reduced overall survival rate [8]. Activation of Wnt/β‐catenin signaling is essential for regulation of cell migration, maintaining stemness of cancer stem cells and colony formation in TNBC cell lines [5]. TNBC with hyperactivated Wnt/β‐catenin signaling is associated with higher grade and is prone to develop lung and brain secondary metastasis [9], which requires that cells of epithelial origin undergo epithelial‐to‐mesenchymal transition (EMT). The EMT process is accompanied by a cadherin switch from the epithelial E‐cadherin (CDH1) to the mesenchymal N‐cadherin (CDH2) and OB‐cadherin (CDH11), loss of polarity and increased migratory capacity [10] [11, 12].

CDH11 is a classical type‐II cadherin that was first identified in mouse osteoblasts [13] and later found to be expressed widely in mesenchymal cell types such as smooth muscle cells, fibroblasts and hair follicle‐derived mesenchymal stem cells [14]. High levels of CDH11 were found in clinical specimens of malignant breast cancer, [15] and increased CDH11 expression in TNBC was correlated with worse overall survival rates [16]. In agreement, both wild‐type and truncated variants of CDH11 were highly expressed in the most invasive TNBC cell line, MDA‐MB‐231, but not in the non‐invasive, non‐triple negative cell line, MCF‐7 [17]. Conversely, overexpression of CDH11 in MCF‐7 increased cell migration and invasion potential significantly [18], whereas disruption of CDH11 function by a neutralizing antibody or knockdown using siRNA significantly reduced tumorigenicity and metastatic potential of TNBC cells [16, 19]. Of note, in addition to breast [17, 18, 20, 21], upregulation of CDH11 expression has been reported in various other tumors including prostate [22, 23], renal [24] and bone [25]. In renal cell carcinoma, CDH11 expression enhanced cell motility and induced high levels of bone metastasis due to the high affinity of homotypic interactions with native osteoblasts [24]. Up‐regulation of CDH11 also promoted advanced prostate cancer metastasis to bone [22]. In contrast, disruption of homotypic CDH11 interactions by an antibody targeting amino acids 343–348 on the CDH11 extracellular EC3 domain reduced the metastatic potential of prostate cancer to bone in a mouse model [25]. Moreover, our laboratory has demonstrated that CDH11 is a key regulator of a diverse set of cellular events including smooth muscle cell contraction [26], proliferation [27] and extracellular matrix (ECM) synthesis [28] that may also be contributing to metastatic potential.

Despite several studies reporting on the involvement of CDH11 in multiple tumors, understanding of the signaling mechanisms underlying the role of CDH11 in tumor growth and metastasis remain elusive. This is the first study reporting the colocalization of CDH11 intracellular domain fragments with β‐catenin in the nucleus, implicating a role of CDH11 in Wnt/β‐catenin signaling. In addition, we discovered that β‐catenin, an intracellular binding partner of CDH11, remains bound to carboxy‐terminal fragments (CTFs) of CDH11 after CDH11 cleavage at the cell surface. CDH11‐CTFs/β‐catenin complex serves to stabilize β‐catenin in the cytoplasm and facilitates its translocation to the nucleus, resulting in activation of Wnt signaling, with subsequent increased proliferation, migration and invasion potential. Our data illuminate a previously unknown role of CDH11 in Wnt/β‐catenin signaling and may facilitate development of strategies to treat TNBC, which is challenging to treat with conventional therapies.

## 2 Materials and methods

### 2.1 Tissue microarray (TMA) and patient cohort

Three tissue microarrays (TMA13, BrCa83, BrCa84) generated by Roswell Park Comprehensive Cancer Center (RPCCC, Buffalo, NY) were utilized for this study. TMA13 contains a total of 209 tumor samples from 206 patients during the period between 1993 and 2010; BrCa83 contains 73 of The Cancer Genome Atlas (TCGA) qualified breast tumor samples and each case has triplicate cores (total of 219 cores) retrieved from 73 patients from 2003 to 2010; and BrCa84 contains 24 primary breast tumor samples with 24 paired metastasis breast tumor cases, each case has triplicate cores (total of 144 cores) isolated from 24 patients during the period between 2003 and 2007. Fresh and frozen tissues were prepared and collected by the tissue procurement lab in RPCCC under remnant tissue protocol I 115707 and tissue microarrays were fabricated using the remnant blocks from the Paraffin Archive Resource (PAR). All samples were obtained following all Ethical and Research code of conducts including the declaration of Helsinki. In general, all samples were collected by the hospital pathology department from patients who have provided written informed consent for the use of their samples. After clinical diagnosis, the remnant samples were formalin fixed and paraffin embedded in a de‐identified manner. Thus, these samples could be provided to investigators for secondary research purposes. According to local legislation, use of these type of samples for research purposes does not fall under the category of research involving human subjects and additional informed consent or issuance of a waiver of consent from an Institutional Review Board (IRB) is not required. Only samples from patients who refused to give a consent were excluded. The BioRepository Bank provides resources to investigators with IRB‐approved protocols (IRB# STUDY00003179 approved by the University at Buffalo IRB).

### 2.2 Automated TMA image analysis

CDH11 protein expression was evaluated in multiple human tumors using a tissue microarray (TMA) containing 209 primary tumor clinical samples retrieved from 206 patients in the period from 1993 to 2010 at RPCCC. To this end, tumor samples were immunostained with an antibody targeting the intracellular domain of CDH11 (Invitrogen, #71‐7600, Rockford, IL, USA) and all immunostained TMAs were scanned by the Aperio™ software at 40× magnification (Fig. S1A,B,H). Surrounding tumor and stroma regions in each core were identified by a pathologist (Dr. Wiam Bshara) with annotation (Fig. S1C,I). Next, QuPath (v0.20‐m8), an open‐source software for quantitative pathology and bioimage analysis [29], was used for quantitative image analysis and spatial localization of CDH11. Briefly, pathologist annotations of tumor and stroma regions were used to train a supervised machine learning model (random forest classifier) to predict the class of the remaining unlabeled cells (Fig. S1D,J). Once cells were labeled as tumor or stromal, the staining intensity of CDH11 was quantitatively compared within each cell compartment – nucleus, cytoplasm, plasma membrane – using an in‐built color deconvolution algorithm (Fig. S1E,F). All measurements were classified using semi‐quantitative scoring, i.e., 0, 1+, 2+, and 3+, corresponding to none, low, medium, and strong staining intensity (Fig. S1G). Two metrics: the H‐score and Allred score were computed. The H‐score is a measure of staining intensity and is defined as a weighted average of staining intensities (3 × %strongly stained cells +2 × %moderately stained cells +1 × %weakly stained cells), ranging from 0 to 300. The Allred score is defined as a combination of two scores: the proportion score, which is based on the percentage of positive cells and ranges from 0 to 5, and the intensity score, which is based on colourimetric intensity and ranges from 0 to 3. The Allred score is used to determine the positive nuclear staining event, where scores in the range 0–2 are considered negative and scores in the range 3–8 are considered positive [30].

### 2.3 Cell culture

Breast cancer cell lines MDA‐MB‐231 (RRID: CVCL_0062), MCF‐7 (RRID: CVCL_0031) and BT‐549 (RRID: CVCL_1092) were obtained from American Type Culture Collection (ATCC, Manassas, VA). Hair‐follicle‐derived stem cell and dermal fibroblast were isolated as described previously [27]. All cells except BT‐549 were cultured in Dulbecco's Modified Eagle medium (DMEM) supplemented with 1% (v/v) antibiotic‐antimycotic cocktail (Thermo Fisher Scientific, Grand Island, NY, USA) and 10% (v/v) fetal bovine serum (Atlanta Biologicals, Norcross, GA, USA). BT‐549 was cultured in RPMI (Thermo Fisher Scientific) supplemented with 1% (v/v) antibiotic‐antimycotic cocktail, 10% (v/v) fetal bovine serum and 0.023 U·mL^−1^ insulin. All cell lines have been authenticated in the past 3 years by STR profiling at ATCC. According to the vendor, the commercially available PowerPlex® 18D kit (Promega, Madison, WI, USA) was used to amplify 17 short tandem repeat (STR) loci plus the gender‐determining locus, Amelogenin. Samples were then processed with the ABI Prism® 3500xl Genetic Analyzer (Thermo Fisher Scientific), and data were analyzed using GeneMapper® ID‐X software (Thermo Fisher Scientific) to confirm cell identity and purity. Finally, all experiments were performed with mycoplasma‐free cells.

### 2.4 Measurement of Wnt activity by TOP‐FLASH assay

The 7TFP plasmid was a gift from Roel Nusse [31]. This plasmid contains a promoter with 7 repeats of TCF regulating firefly luciferase expression, and the expression level of luciferase directly reflects the Wnt pathway activity. To measure the Wnt pathway activity, MDA‐MB‐231 + 7TFP cell line was generated by transducing MDA‐MB‐231 with a lentivirus encoding for the 7TFP vector followed by selection with puromycin. Next, cell lysates were harvested by using the Pierce Firefly Luciferase Flash Assay Kit (Thermo Fisher Scientific, #16177) according to the protocol. Finally, firefly luciferase activity was determined by a plate reader (Synergy 4; Biotek, Winooski, VT, USA) after 30 min incubation with luciferin.

### 2.5 Western blot

Cell lysates were harvested from cell monolayers using a lab‐made standard lysis buffer (20 mM Tris–HCl pH 8.0, 137 mM NaCl, 2 mM EDTA, 10% [v/v] Glycerol, 1% [v/v] Triton X‐100) supplemented with a cocktail of protease inhibitors (Thermo Fisher Scientific), 1× blue loading buffer (Cell Signaling Technology, Danvers, MA, USA) and 1x DTT (Cell Signaling Technology). In some cases, to enhance the levels of CTFs, cell lysates were prepared by first detaching cells from the surface with TrypLE Express (ThermoFisher Scientific) for 1–2 min, washed once with PBS, before the addition of lysis buffer. Proteins in cell lysates were separated in precast 10% Tris–glycine gels (Thermo Fisher Scientific) by electrophoresis and subsequently transferred to PVDF membranes (Millipore, Billerica, MA, USA) by the Trans‐Blot Turbo system (Bio‐Rad, Hercules, CA, USA). Primary antibodies (Table S1) were incubated with the membranes at 4 °C overnight, which was followed by HRP‐conjugated secondary antibody (Table S1) incubation at room temperature for 1 h. Protein bands were developed with LumiGLO reagent (Cell Signaling Technology) according to the manufacturer's protocol and detected using a ChemiDoc Imaging System (Bio‐Rad). Protein content was analyzed by densitometric analysis using the NIH image j software (RRID:SCR_003070, 1.48v) and normalized to GAPDH.

### 2.6 Protein subcellular fractionation and Co‐Immunoprecipitation

Proteins from different subcellular compartments including cytoplasmic, nuclear, chromatin‐bound, membrane and cytoskeletal were extracted by using the subcellular protein fraction kit (Thermo Fisher Scientific, #78840) following the manufacturer's protocol and separated protein lysates were subjected to western blotting to visualize protein distribution within each subcellular compartment. Co‐Immunoprecipitation was performed using Pierce Co‐Immunoprecipitation Kit (Thermo Fisher Scientific, #26149) per manufacturer's instruction. Pulled‐down lysates were subjected to western blotting for detection of target protein complex.

### 2.7 Generation of CDH11 knockout MDA‐MB‐231 cell line by CRISPR‐CAS9

lentiCRISPR‐ v2 was a gift from Feng Zhang [32] and used for the generation of CDH11 knockout MDA‐MB‐231 cell line. Guide RNA with a sequence TTGACAATGAATTCCGACGGTGG targeting CDH11 was designed using CHOPCHOP (RRID:SCR_015723, https://chopchop.cbu.uib.no/). MDA‐MB‐231 was transfected with lentiCRISPR‐sgCDH11 vector by using Lipofectamine 3000 (Thermo Fisher Scientific, #L3000001) and a clonal assay was performed to obtain CDH11 knockout cell line. Knockout CDH11 expression in MDA‐MB‐231 was verified by sequencing, PCR and western blot.

### 2.8 qRT‐PCR

Quantitative reverse transcription polymerase chain reaction was performed as described previously [27]. Briefly total mRNA was extracted using the RNeasy Mini Kit (Qiagen, Valencia, CA, USA) according to the manufacturer's protocol and single‐strand cDNA was synthesized using High Capacity cDNA Reverse Transcription Kit (Applied Biosystems, Foster City, CA, USA, #4368814). Next, cDNA was amplified with primer pairs as shown in Table S2 and the Power SYBR™ green PCR Master kit (Applied Biosystems, #4367659) in a CFX96 real‐time PCR detection system (Bio‐Rad). Gene expression levels were quantified and analyzed using the ΔCT method and reported after normalization with the corresponding expression level of the housekeeping gene, *GAPDH*.

### 2.9 Immunofluorescence staining

Immunofluorescence staining was performed as described previously [27]. Briefly cells at the indicated conditions were fixed with 4% (w/v) paraformaldehyde for 15 min at room temperature; permeabilized with 0.1% (v/v) Triton‐X100 in PBS (10 min, room temperature); and incubated in blocking buffer (5% [v/v] goat serum in PBS/0.1% [v/v] Triton‐X100) at room temperature for 1 h. The cells were then incubated with primary antibodies (Table S1) overnight at 4 °C followed by three washes with PBS. Subsequently, samples were incubated with a secondary antibody (Table S1) at room temperature for 1 h. Alexa Fluor 594 Phalloidin (Thermo Fisher Scientific) was used to stain for F‐actin as per manufacturer's instructions. Cell nuclei were counterstained with Hoechst 33342 (10 mg·mL^−1^; 1 : 200 dilution in PBS; 5 min at room temperature; Thermo Fisher Scientific). Stained cells were visualized with a Zeiss AxioVision imaging system (RRID:SCR_002677, Observer Z1 LSM 510 microscope; Zeiss, Oberkochen, Germany) equipped with a digital camera (ORCA‐ER C4742‐80; Hamamatsu, Bridgewater, NJ, USA) and fluorescent images were quantitatively analyzed using the NIH image j software.

### 2.10 Matrigel invasion assay

Corning Matrigel Basement Membrane Matrix (Corning, Glendale, AZ, USA) was diluted to 200–300 μg·mL^−1^ in 100 μL coating buffer (0.01 m Tris, 0.7% [w/v] NaCl, pH = 8.0) and allowed to polymerize in a 96 well size, 8‐μm pore size transwell insert (VWR, Radnor, PA, USA) for 2 h at 37 °C and the residual coating buffer was removed at the end of incubation, without disturbing the layer of Matrigel matrix on the membrane. Five hundred thousand cells suspended in 100 μL of serum‐free medium were seeded in the upper chamber and 500 μL of chemoattractant (DMEM with 10% [v/v] FBS) were added to the lower chamber. After 24 h of incubation, cells were fixed in 4% (w/v) paraformaldehyde and monolayer of cells on the top side of the insert was removed to reduce background. Cells that transmigrated through the insert were stained with Hoechst 33342. Images of invaded cells on the membrane were taken, and percent invasion was calculated based on the following formula: (number of cells moving through the inserts/number of cells seeded) × 100.

### 2.11 Wound healing assay

Thirty thousand cells per well were seeded in the ibidi 2‐well insert (ibidi, Fitchburg, WI, USA) that was placed in a 24‐well plate and after 48 h incubation, the insert was removed, leaving a 500 μm gap between the two separate cell monolayers. Afterwards, a 24 h time‐lapse video was recorded with a time interval of 1 h. Each image was analyzed by imagej software to measure the area occupied by cells and wound area, which were used to calculate % wound area and % healed area.

### 2.12 Statistical analysis

Values are means ± SD. Significant differences between groups were determined by Student's *t*‐test (paired, two‐tailed) or ANOVA (Analysis of Variance) with post hoc analysis using Student–Newman–Keuls multiple comparison test or Spearman correlation analysis.

## 3 Results

### 3.1 Cadherin‐11 is expressed in multiple types of human cancers

**Fig. 1:**
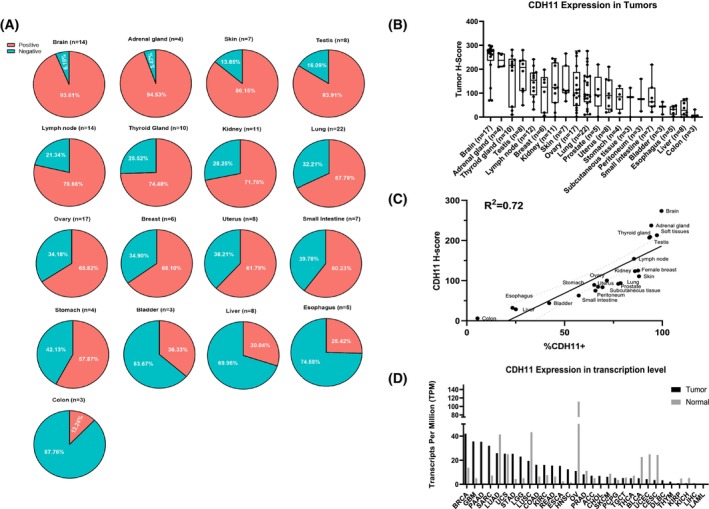
Tissue microarray (TMA) analysis reveals CDH11 expression in various tumors, including breast cancer from patients. (A) The percentage of CDH11^+^ cells and (B) CDH11 protein expression level as indicated by H‐score in the tumors were assessed by QuPath based on immunohistochemistry of tumor microarrays containing 209 tumors retrieved from 206 patients. Tissues (*n* < 3) are not listed. (B) Data values are shown in a boxplot with the line in the box representing the median and the upper and lower ends of the whisker indicating the maximum and minimum value, respectively. (C) A linear correlation was detected between the percentage of CDH11^+^ in tumors and CDH11 expression level with *R* ^2^ = 0.72. (D) Transcriptional level of CDH11 expression in different tumor types when compared with normal tissue control from TCGA database, plotted by GEPIA; ACC, adrenocortical carcinoma; BLCA, bladder urothelial carcinoma; BRCA, breast invasive carcinoma; CESC, cervical squamous cell carcinoma and endocervical adenocarcinoma; CHOL, cholangio carcinoma; COAD, colon adenocarcinoma; DLBC, lymphoid neoplasm diffuse large B‐cell lymphoma; ESCA, esophageal carcinoma; GBM, glioblastoma multiforme; HNSC, head and neck squamous cell carcinoma; KICH, kidney chromophobe; KIRC, kidney renal clear cell carcinoma; KIRP, kidney renal papillary cell carcinoma; LAML, acute myeloid leukemia; LGG, brain lower grade glioma; LIHC, liver hepatocellular carcinoma; LUAD, lung adenocarcinoma; LUSC, lung squamous cell carcinoma; OV, ovarian serous cystadenocarcinoma; PAAD, pancreatic adenocarcinoma; PCPG, pheochromocytoma and paraganglioma; PRAD, prostate adenocarcinoma; READ, rectum adenocarcinoma; SARC, sarcoma; SKCM, skin cutaneous melanoma; STAD, stomach adenocarcinoma; TGCT, testicular germ cell tumors; THCA, thyroid carcinoma; THYM, thymoma; UCEC, uterine corpus endometrial carcinoma; UCS, uterine carcinosarcoma.

To assess CDH11 protein expression in human tumors from various anatomical locations, we employed a tissue microarray (TMA) containing 209 primary tumor clinical samples and immunostained for the intracellular domain of CDH11. H‐score was then computed based on the intensity of CDH11 staining in each tumor sample (see Section 2 for details). Out of these tumor samples, we found a high percentage of CDH11^+^ tumor cells in multiple tissues including brain (93.8%, *n* = 14), adrenal gland (94.5%, *n* = 4), skin (86.2%, *n* = 7), testis (83.9%, *n* = 8), lymph nodes (78.7%, *n* = 14), thyroid gland (74.5%, *n* = 10) and kidney (71.8%, *n* = 11); while lower percentage of CDH11^+^ tumor cells was detected in the liver (30.0%, *n* = 8), esophagus (25.4%, *n* = 5) and colon (12.2%, *n* = 3) (Fig. 1A). In addition, a strong signal intensity of CDH11 (median H‐score above 200) was observed and quantitatively reported in tumors from brain (*n* = 17, median score (M.S.) of 273), adrenal gland (*n* = 4, M.S. = 237), thyroid gland (*n* = 10, M.S. = 216) and testis (*n* = 8, M.S. = 207); medium signal intensity of CDH11 (median H‐score between 100 and 200) was measured in tumors originating from lymph node (*n* = 12, M.S. = 154), breast (*n* = 6, M.S. = 125), kidney (*n* = 11, M.S. = 124), skin (*n* = 7, M.S. = 111) and ovary (*n* = 17, M.S. = 100); while weak CDH11 signal intensity (median H‐score below 100) was detected in tumors from lung (*n* = 22, M.S. = 94), prostate (*n* = 5, M.S. = 92), uterus (*n* = 8, M.S. = 89), stomach (*n* = 4, M.S. = 95), subcutaneous tissue (*n* = 3, M.S. = 84), peritoneum (*n* = 3, M.S. = 75), small intestine (*n* = 7, M.S. = 64), bladder (*n* = 3, M.S. = 44), esophagus (*n* = 5, M.S. = 32), liver (*n* = 8, M.S. = 29) and colon (*n* = 3, M.S. = 6) (Fig. 1B). In general, tumor cells exhibiting high CDH11 expression, as indicated by high H‐score, are associated with high percentage of CDH11 positive cells in the tumor region (*R* ^2^ = 0.78) (Fig. 1C).

In addition, examination of CDH11 expression based on tumor types (Fig. S1K) revealed high expression level of CDH11 in astrocytoma (*n* = 3, M.S. = 278), glioblastoma (n = 5, M.S. = 277), oligodendroglioma (*n* = 3, M.S. = 264), meningioma (*n* = 6, M.S. = 248), pheochromocytoma (*n* = 4, M.S. = 237) and mesothelioma (*n* = 2, M.S. = 235). Medium expression level of CDH11 was observed in sarcoma (*n* = 2, M.S. = 171), seminoma (*n* = 4, M.S. = 164), lymphoma (*n* = 15, M.S. = 145), teratoma (*n* = 3, M.S. = 114) and carcinoma (*n* = 56, M.S. = 110). Low expression of CDH11 was observed in melanoma (*n* = 6, M.S. = 100), adenocarcinoma (*n* = 49, M.S. = 81) and carcinoid tumors (*n* = 14, M.S. = 72).

Next, we used the online platform GEPIA [33] (Gene Expression Profiling Interactive Analysis) to analyze The Cancer Genome Atlas (TCGA) RNA‐seq data, in terms of the transcription levels of *CDH11* across all tumor samples and compared them with paired normal tissues. Fold changes were quantified using the limma methodology computed by GEPIA platform [34]. In agreement with the TMA data, *CDH11* was highly expressed in cancerous tissues from breast (*n* = 1085, M.S. = 41.97), brain (*n* = 163, M.S. = 35.56) and lung (*n* = 483, M.S. = 25.83). In addition, *CDH11* expression level was up‐regulated by > 3‐fold (*P* < 0.05) in breast, > 7‐fold in brain (*P* < 0.05), > 39‐fold in pancreatic (*P* < 0.05) and 110‐fold in lymphoma (*P* < 0.05) cancers when compared with the corresponding normal tissues (Fig. 1D).

### 3.2 Cadherin‐11 is localized in the nuclei of multiple human tumors

**Fig. 2:**
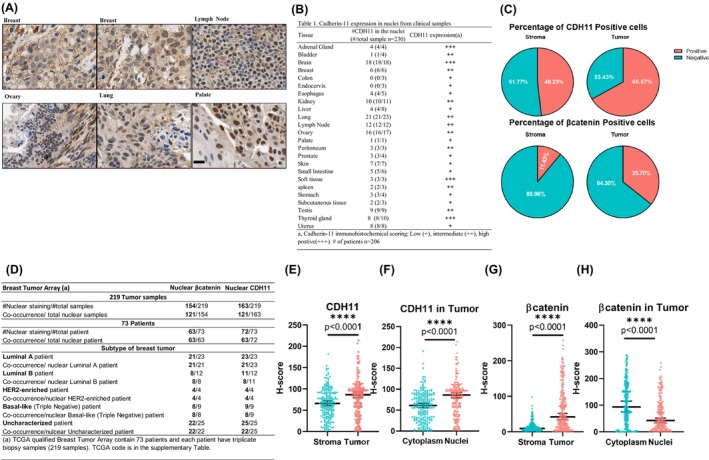
Assessment of nuclear CDH11 expression in different tumor types. (A) Representative immunohistochemistry images display the presence of nuclear CDH11 in breast (*n* = 6), lymph node (*n* = 12), ovary (*n* = 17), lung (*n* = 22) and palate (*n* = 2) cancer, scale bar = 20 μm. (B) Table showing the presence of nuclear CDH11 in the CDH11‐expressing tumors, *n* = 230 samples from 206 patients. (C) Pie charts showing the percentage of CDH11^+^ and β‐catenin^+^ cells in breast cancer tumor/stroma regions based on the analysis from 219 breast tumor samples. (D) Table summarizing the event of nuclear CDH11 and nuclear β‐catenin from 219 breast tumor samples retrieved from 206 patients. (E–H) QuPath analysis of CDH11 and β‐catenin from a breast tumor cancer TMA (*n* = 206), Data values are shown as median ± SEM. (E, G) H‐score of CDH11 and β‐catenin expression level was individually assessed in the stroma region and tumor region. (F, H) Within the tumor region, the expression level of CDH11 and β‐catenin in the nuclei and cytoplasm was individually characterized and reported as H‐score.

Surprisingly, while CDH11 is a junctional protein, it was found to localize in the nuclei of tumor cells from multiple tumors including breast, lymph node, ovary, lung and palate (Fig. 2A). In particular, the majority of cancers including breast (6/6), brain (18/18), adrenal gland (4/4), lung (21/23), lymph node (12/12), ovary (16/17) and testis (9/9) exhibited high occurrence of nuclear CDH11 (Fig. 2B). Interestingly, no nuclear CDH11 was detected in colon (0/3) and endocervix (0/3), where the levels of CDH11 expression were low. Medium levels of nuclear CDH11 were seen in the bladder, breast, kidney, lung, lymph node, ovary, peritoneum and spleen (Fig. 2B).

### 3.3 Increased expression and nuclear localization of CDH11 in breast tumors

Breast cancer is the most common cancer diagnosed globally [35] and the increased CDH11 expression detected in TNBC correlates with malignant breast cancer [15, 17] and worse survival rates. [36] In addition to high CDH11 expression (Fig. 1D) and nuclear localization in breast cancer tissue samples (Fig. 2A,B), analysis of TMA samples revealed significantly elevated expression of CDH11 in breast cancer tissues of all four stages when compared to normal tissue (Fig. S2A). Collectively, these clinical data prompted us to examine the biological functions of nuclear CDH11 in breast cancer more closely.

To this end, we employed a breast cancer‐specific TMA containing 219 primary breast tumors retrieved from 73 patients in the period between 2003 and 2010. In agreement with the larger multi‐tumor array, the percentage of CDH11^+^ cells in these two tumor arrays was highly similar (breast cancer TMA: % CDH11^+^ cells = 66.6, *n* = 219 vs. multiple tissue TMA: %CDH11^+^ cells = 65.8, *n* = 6). Moreover, the percentage of CDH11^+^ cells in the tumor areas was significantly higher when compared with the surrounding stroma (tumor: 66.6% vs. stroma: 48.2%, *n* = 219, *P* < 0.001). In addition, the percentage of β‐catenin^+^ cells was significantly higher in the tumor region when compared with surrounding stroma (tumor: 35.7% vs. stroma: 11.4%, *n* = 219 *P* < 0.001; Fig. 2C).

Aberrant Wnt/β‐catenin pathway activation results in the accumulation of β‐catenin in the nucleus and contributes to tumorigenesis in the breast. Given that β‐catenin is a classical CDH11 intracellular binding partner at the cell surface [37], we investigated whether β‐catenin and CDH11 co‐localized in the nucleus of cells in the breast tumor clinical samples. Remarkably, the vast majority of breast cancer samples exhibited nuclear β‐catenin (86.3%, or 63/73) and nuclear CDH11 (98.6%, or 72/73; Fig. 2D). Moreover, all 63 patient samples exhibiting nuclear β‐catenin were found to contain nuclear CDH11 (63/63). Conversely, 87.5% (63/72) of nuclear CDH11‐positive breast cancer patient samples were also positive for nuclear β‐catenin. This high co‐occurrence rate indicated a potential correlation between the two events. Moreover, breast tumor cells exhibited an elevated CDH11 expression level when compared with the surrounding stroma cells (tumor M.S. = 86.4 vs. stroma M.S. = 65.9, *n* = 219, *P* < 0.0001; Fig. 2E). Within the breast tumor cells, a higher CDH11 level was detected in the nucleus when compared with the cytoplasm (nucleus M.S. = 86.38 vs. cytoplasm M.S. = 61.28, *n* = 219, *P* < 0.0001; Fig. 2F). Similar trend was observed for β‐catenin: tumor cells expressed four‐fold higher level of β‐catenin when compared with stroma cells (tumor M.S. = 63.47 vs. stroma M.S. = 14.67, *n* = 219, *P* < 0.0001; Fig. 2G), with 37.5% of total β‐catenin localized in the nucleus (nucleus M.S. = 63.46 vs. cytoplasm M.S. = 104.86, *n* = 219, *P* < 0.0001; Fig. 2H, Fig. S2B–E).

### 3.4 Co‐localization of CDH11 and β‐catenin in the nucleus of breast cancer cells

**Fig. 3:**
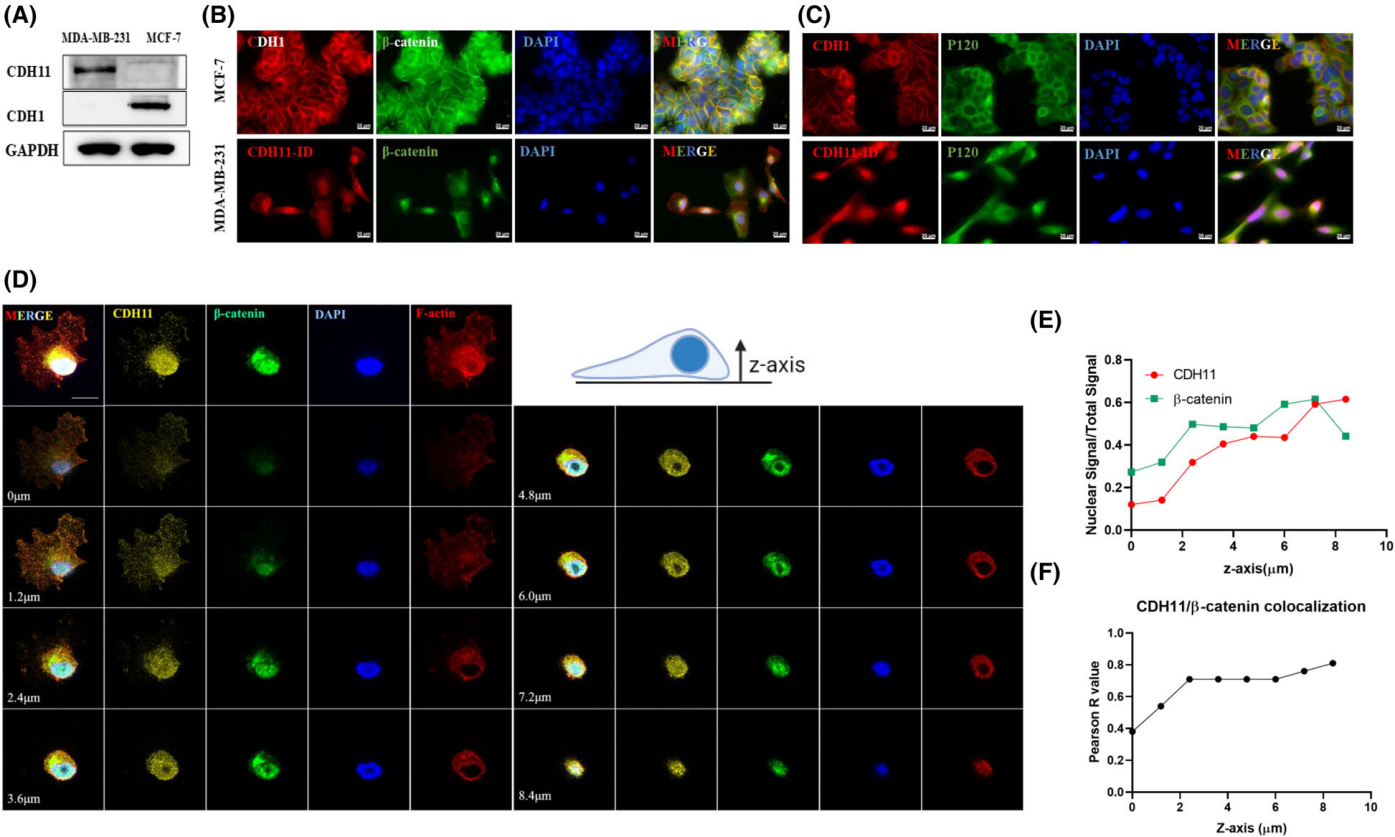
Spatial distribution of CDH11 and β‐catenin in breast cancer cell lines. (A) Representative western blot detecting CDH11, CDH1 and GAPDH in MDA‐MB‐231 and MCF‐7 cell lines. (B, C) Representative immunostaining images visualizing CDH1, CDH11, β‐catenin and P120 spatial distribution in MDA‐MB‐231 and MCF‐7, scale bar = 20 μm. (D) Single‐cell z‐stack confocal images display nuclear CDH11 co‐localizing with nuclear β‐catenin. Anti‐CDH11 antibody targeting the intracellular domain of CDH11 was used to detect CDH11, scale bar = 20 μm. Nuclear CDH11 and β‐catenin signaling in the nucleus was quantified in (E) and co‐localization relationship between CDH11 and β‐catenin was quantified in (F). Data shown was a representative of *n* = 3 experiments.

Next, we compared two well‐characterized ductal breast carcinoma cell lines, MCF‐7 and MDA‐MB‐231. MCF7 are hormone‐dependent cells, grow as epithelial colonies and express E‐cadherin (CDH1); while MDA‐MB‐231 are triple negative cells, with limited cell–cell contact and express CDH11 (Fig. 3A). In both cell lines, β‐catenin co‐localized with the corresponding cadherin. Specifically, in MCF‐7 cells, the majority of β‐catenin co‐localized with CDH1 at the intercellular junctions. On the other hand, in MDA‐MB‐231 cells, β‐catenin was concentrated in the nucleus co‐localizing with CDH11 (Fig. 3B). In contrast, p120, another intracellular cadherin‐binding partner, remained mostly on the cell surface in both cell lines (Fig. 3C).

To better visualize the spatial co‐localization of β‐catenin and CDH11, high‐resolution confocal microscopy was employed to image single MDA‐MB‐231 cells and generate 3‐dimensional z‐stack images (Fig. 3D, Fig. S3). Indeed, both β‐catenin and CDH11 were found in the cytoplasm and nucleus, as seen by the z‐stacks where both signal intensities increased with the height, z, reaching a plateau within the range of 4–8 μm, which is where the nucleus is located (Fig. 3E). In agreement, the Pearson correlation also increased with increasing z to about 0.8 (Fig. 3F), suggesting co‐localization of β‐catenin with CDH11 throughout different cellular compartments including in nucleus.

### 3.5 CDH11 intracellular domain is localized in the cell nucleus

**Fig. 4:**
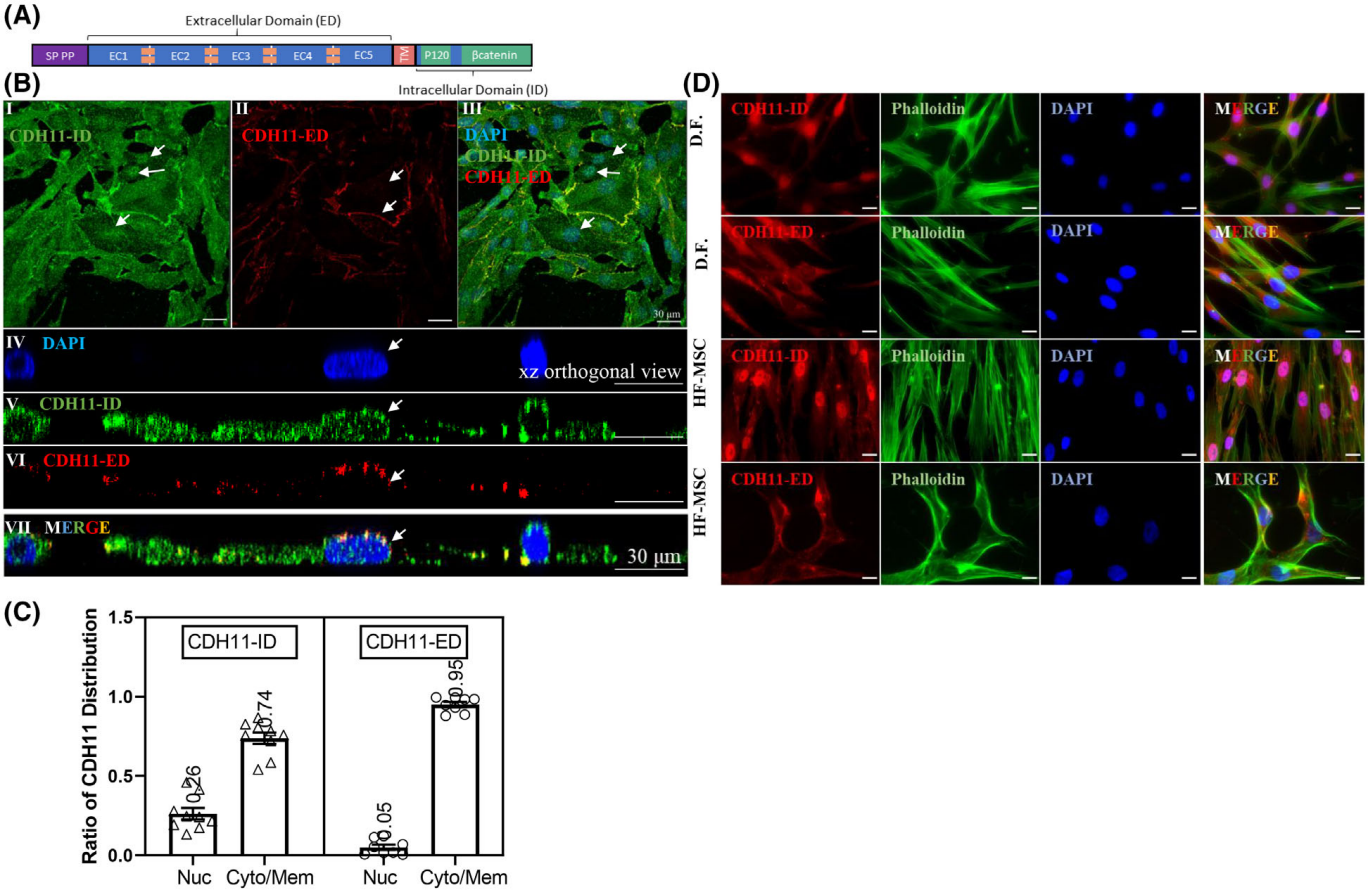
Intracellular domain but not extracellular domain of CDH11 is found in the cell nuclei. (A) A schematic demonstrating the structure of CDH11, ED, extracellular domain; ID, intracellular domain; P120, p120 binding domain; PP, pro‐peptide; SP, signaling peptide; TM, transmembrane; β‐catenin, β‐catenin binding domain. (B) I, II, III: Representative 2D confocal images display a distinct spatial distribution pattern of CDH11 intracellular domain (ID) localized throughout the cells including the nuclei (I, III), while CDH11 extracellular domain (ED) was localized at the cell periphery (II, III) in MDA‐MB‐231, scale bar = 30 μm; Orthogonal xz view of z‐stack confocal image exhibit CDH11‐ID localized in the nuclei (IV, V, VII) and CDH11‐ED localized at the cell periphery (IV, VI, VII). Arrows indicate the nuclei, scale bar = 30 μm. (C) The spatial distribution of CDH11‐ID and CDH11‐ED was quantified in terms of nuclear intensity and cytoplasmic/membrane intensity. Results are shown as mean ± SD. (D) Representative fluorescent immunostaining detecting CDH11‐ID, CDH11‐ED, phalloidin, and nuclei (DAPI) in CDH11‐expressing cell types: dermal fibroblast (D.F.) and hair follicle‐derived mesenchymal stem cells (HF‐MSC), scale bar = 20 μm. Data shown are representative of *n* = 3 experiments.

Next, we utilized two anti‐CDH11 antibodies against either the intracellular domain (ID) or the extracellular domain (ED) to track the spatial location of CDH11 in different cell compartments (Fig. 4A). Interestingly, the intracellular domain of CDH11 was localized at the adherens junctions but mostly in the cytoplasm and nucleus (Fig. 4B,C, Fig. S4). On the other hand, the extracellular domain of CDH11 was predominantly localized at the adherens junctions, as expected (Fig. 4B,C). This data may suggest that a cleavage event might occur leading to dissociation of CDH11‐ID from the full‐length CDH11 and translocation to the nucleus.

We also examined two other CDH11‐expressing mesenchymal cell types: dermal fibroblasts (DF) and hair follicle‐derived mesenchymal stem cells (HF‐MSC). Similar to tumor cells, we observed nuclear localization of CDH11‐ID in both mesenchymal cell types (Fig. 4D), suggesting that CDH11 cleavage and nuclear translocation of CDH11‐ID may not be restricted only to tumor cells.

### 3.6 CDH11 is necessary to maintain active β‐catenin and Wnt pathway activity

**Fig. 5:**
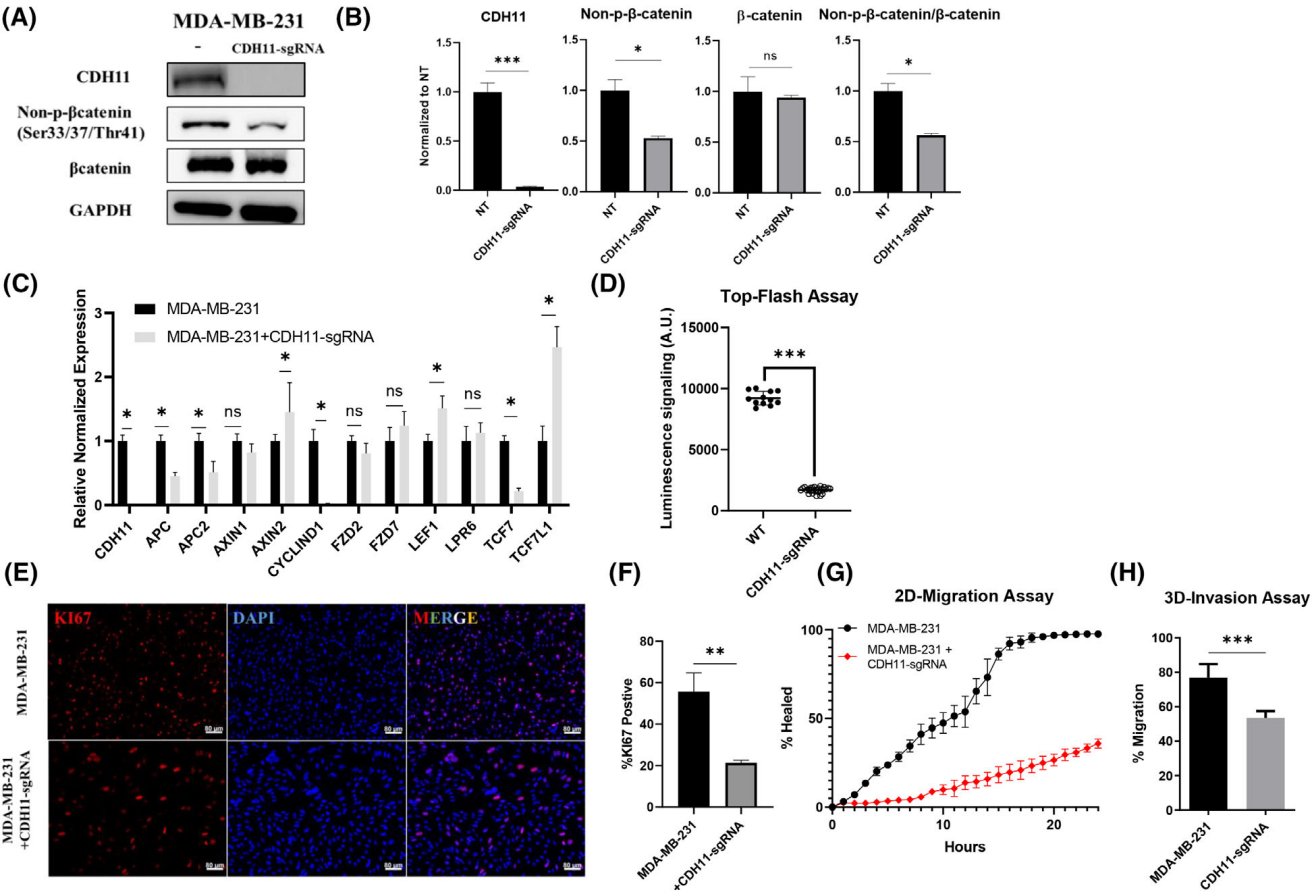
Loss of CDH11 decreases Wnt pathway activity and undermines metastasis potential of triple‐negative breast cancer cell. (A) Knock out (KO) *CDH11* gene in MDA‐MB‐231 via CRISPR/Cas9 and western blot detecting CDH11, non‐phosphorylated β‐catenin (Ser33/37/Thr41), total β‐catenin and GAPDH in MDA‐MB‐231 wild‐type (WT) and KO cells. (B) The protein expression levels in (A) were quantified. (C) mRNA level of Wnt pathway associated genes were measured by quantitative real‐time PCR including APC, APC2, AXIN1, AXIN2, CYCLIND1, FZD2, FZD7, LEF1, LPR6, TCF7, TCF7L1 as well as CDH11. (D) Wnt pathway activity was assessed by Top‐Flash assay in WT and CDH11 KO cell lines. (E) Immunostaining detecting KI67 in MDA‐MB‐231 and CDH11 KO cell lines, scale bar = 50 μm. (F) Percentage of KI67^+^ in (E) was quantified. (G) 2D wound healing assay is performed to examine the migration rate of MDA‐MB‐231 and CDH11 KO cells and percentage of the wound healed within 24 h was reported (*n* = 3). (H) 3D invasion assay was performed to measure the invasion capacity of MDA‐MB‐231 and CDH11 KO cells and the percentage of migrated cells through the matrigel‐coated insert was reported after 24 h (*n* = 3). Results are shown as mean ± SD. * denotes *P* < 0.05, ** denotes *P* < 0.005, *** denotes *P* < 0.0005 when compared with corresponding control (unpaired two‐tailed Student's *t*‐test). Data shown are representative of *n* = 3 experiments.

These results prompted us to hypothesize that CDH11 might be required for the activation of the β‐catenin/Wnt pathway. To address this hypothesis, we knocked out *CDH11* in the MDA‐MB‐231 cell line via CRISPR/Cas9, resulting in the insertion of 117 bp sequence including a termination codon and successful knock‐out (KO) was verified at the protein and mRNA level (Fig. 5A–C). This loss of CDH11 resulted in a two‐fold reduction of non‐phosphorylated (active) β‐catenin, with no effect on the pool of total β‐catenin (Fig. 5A,B). While upstream genes that are known to regulate the Wnt pathway, e.g., FZD2, FZD7 and LPR6 co‐receptors or AXIN1/2, showed no significant or small change, expression of cyclin D1, a direct Wnt target gene, was diminished in KO cells (Fig. 5C). In agreement, the Top‐Flash assay revealed that loss of CDH11 abolished Wnt pathway activity (WT = 9205 A.U., KO = 1684 A.U., *P* < 0.001; Fig. 5D) and resulted in a significant reduction of proliferation as evidenced by the decrease in Ki67^+^ cells from 60% in WT to 20% in CDH11 KO cells (Fig. 5E,F). In addition, loss of CDH11 impaired the migratory capacity of tumor cells as shown using a 2D wound healing assay. While WT cells were highly migratory closing the 2D wound in around 20 h, CDH11 KO cells failed to close half the wound area even after 24 h (Fig. 5G). Finally, the transwell invasion assay showed that cell invasion through Matrigel‐coated inserts decreased from 78% in WT cells to 50% in CDH11 KO cells, suggesting a significant loss of invasion potential (Fig. 5H).

### 3.7 Inhibiting CDH11 cleavage reduces Wnt activity and cancer cell proliferation

Next, we examined whether cleavage of CDH11 affected β‐catenin phosphorylation, thereby increasing the amount of active β‐catenin in the nucleus and Wnt activation. First, we employed an antibody against the CDH11‐ID to see whether CDH11 is cleaved and releases the CDH11‐ID. Interestingly, one full‐length (FL) CDH11 (110 kDa) and three small C‐terminal fragments (CTF1/2/3) with corresponding MW of ~ 49, 40 and 34 kDa were detected, indicating a series of CDH11 cleavage events in MDA‐MB‐231 and another TNBC cell line, BT‐549 (Fig. S5).

**Fig. 6:**
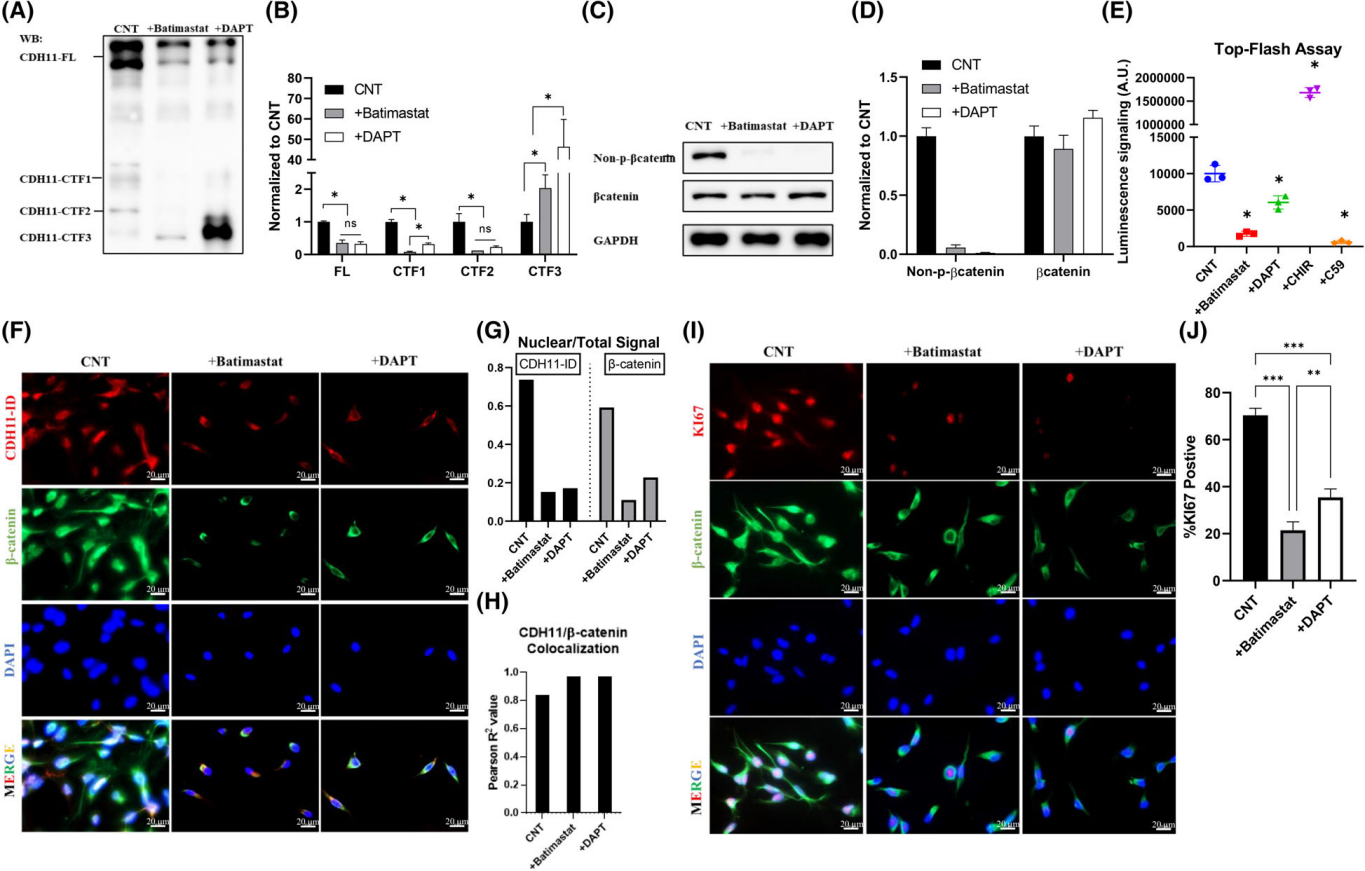
Interference of CDH11 cleavage machinery leads to a reduction of Wnt pathway activity in triple‐negative breast cancer cell line. (A)Western blot detecting CDH11‐FL and CDH11‐CTF1/2/3 after inhibition of CDH11 cleavage machinery by ADAMs inhibitor (50 nm of batimastat) and γ‐secretase inhibitor (2 μm of DAPT) for 2 days. The corresponding band intensities were quantified in (B). (C) Non‐p‐β‐catenin, total β‐catenin and GAPDH were detected by western blot after exposure to ADAMs and γ‐secretase inhibitors (50 nm of batimastat, 2 μm of DAPT) for 2 days. Band intensities were quantified in (D). CNT: Control sample without treatment. (E) Wnt pathway activity was assessed by Top‐Flash assay in the presence of batimastat (50 nm), DAPT (2 μm), CHIR99021 (CHIR; 5 μm) or Wnt‐C59 (C59; 2 μm) for 2 days. (F) Immunostaining for CDH11‐ID and β‐catenin in the presence of batimastat (50 nm) or DAPT (2 μm), scale bar = 20 μm. The corresponding nuclear/total fluorescence signals were quantified in (G); Co‐localization analysis of CDH11 and β‐catenin was performed, and Pearson *R* value was reported in (H); (I) Immunofluorescence staining visualizes KI67, β‐catenin and GAPDH in MDA‐MB‐231 after 2 days treatment with batimastat (50 nm) or DAPT (2 μm), scale bar = 20 μm. (J) Percentage of KI67‐positive cells in (I) was quantified. Results are shown as mean ± SD. * denotes *P* < 0.05, ** denotes *P* < 0.005, *** denotes *P* < 0.0005 when compared with corresponding control (unpaired two‐tailed Student's *t*‐test). Data shown are representative of *n* = 3 experiments.

Subsequently, we employed inhibitors of ADAMs (batimastat) and γ‐secretase (DAPT), two proteases that were shown to act sequentially to generate CDH11‐CTF1 and CTF2 [38, 39]. These inhibitors did not affect cell viability as evidenced by live/dead assay (Fig. S6A). Interestingly, blocking ADAMs or γ‐secretase reduced the amount of FL CDH11 by ~ 70%; batimastat decreased CDH11‐CTF1 and CTF2 to a minimal level of 8% and 12% of control, respectively, while DAPT had a similar effect reducing CDH11‐CTF1 and CTF2 to 30% and 20%, respectively (Fig. 6A,B). Of note, blocking ADAMs or γ‐secretase resulted in high levels of CDH11‐CTF3, which may be targeted to degradation, as previously reported [38].

Both inhibitors diminished non‐phosphorylated (active) β‐catenin without affecting total β‐catenin levels (Fig. 6C,D). In agreement, batimastat and DAPT decreased the nuclear accumulation of CDH11‐ID to 15% and 17% and β‐catenin to 11% and 22% (Fig. 6F,G), even though CDH11 and β‐catenin remained highly co‐localized (*R* ^2^ = 0.97, Fig. 6F,H). As a result, batimastat diminished Wnt activity to a similar level as Wnt inhibitor C59 or CDH11 KO; while DAPT reduced it significantly but to a lesser extent (Fig. 6E). In accordance, batimastat and DAPT decreased proliferation significantly as evidenced by the %Ki67^+^ cells (21% with batimastat, 35% with DAPT) when compared with control (70%) (Fig. 6I,J, Fig. S6B).

### 3.8 CDH11 C terminal fragments (CTFs) translocate to the nucleus with β‐catenin

**Fig. 7:**
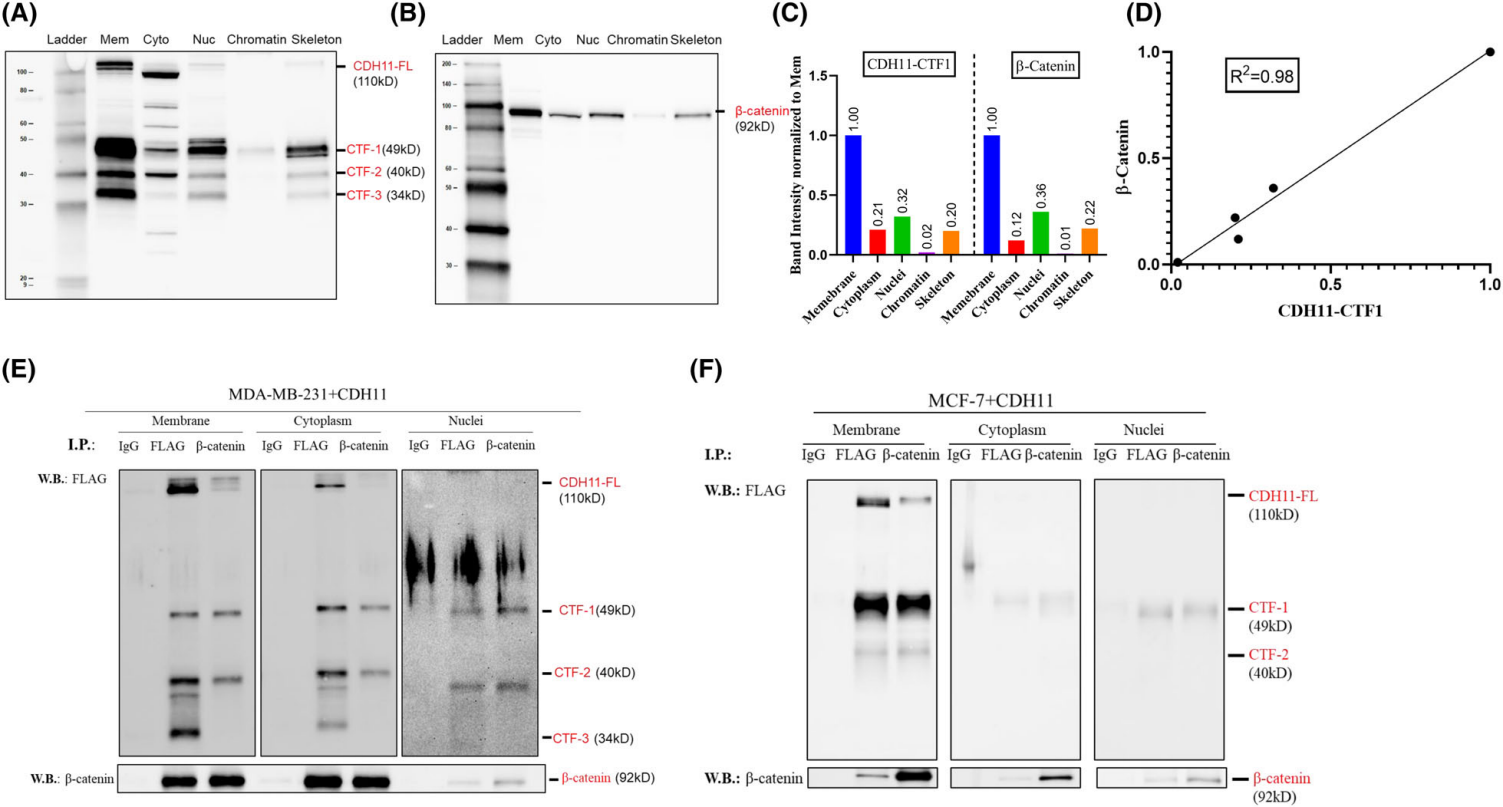
β‐catenin translocates to the nuclei in the complex of CDH11‐CTFs/β‐catenin. (A) Subcellular fractionation western blot revealing the distribution pattern of full‐length CDH11 (CDH11‐FL) and CDH11‐CTFs in different subcellular fractions including membrane, cytoplasmic, nuclear, chromatin‐bound and cytoskeleton associated proteins. (B) Subcellular fractionation western blot detecting β‐catenin distribution within each subcellular compartment. (C) Quantification of CDH11‐CTF1 and β‐catenin distribution within each subcellular compartment from western blot data and a linear correlation between these 2 molecules was reported in (D) with *R* ^2^ = 0.98. (E, F) Overexpression of FLAG‐tagged CDH11 in MDA‐MB‐231 and MCF‐7 by viral transduction to generate MDA‐MB‐231 + CDH11 and MCF‐7 + CDH11 cell lines, respectively. Subcellular protein lysates were extracted and co‐immunoprecipitated with antibodies against FLAG and β‐catenin followed by western blot detection for FLAG and β‐catenin. Data shown was representative of *n* = 3 experiments.

Next, we investigated the distribution of FL CDH11 and CDH11‐CTFs in different cellular compartments of MDA‐MB‐231 cells, including membrane, cytoplasm, nucleus, chromatin and cytoskeleton. Surprisingly, we observed much higher levels of CDH11‐CTFs in these experiments, which require cell detachment prior to sequential fractionation by differential centrifugation. As expected, FL CDH11 and CDH11‐CTFs were found to be predominantly associated with the cell membrane fraction (Fig. 7A). Among all CDH11‐CTFs, CDH11‐CTF1 was found in the nucleus at higher levels when compared with CDH11‐CTF2 and CDH11‐CTF3 (Fig. 7A), exhibiting similar distribution pattern as β‐catenin (Fig. 7B). Indeed, the levels of CDH11‐CTF1 and β‐catenin in each compartment correlated very closely (*R* ^2^ = 0.98) (Fig. 7C,D), prompting us to hypothesize that CDH11‐CTF1 and β‐catenin might be translocated to the nucleus as a complex.

Indeed, forced expression of CDH11 that was fused with FLAG tag at the C‐terminus (FLAG) in MCF‐7 cells lacking endogenous CDH11 led to increased accumulation of β‐catenin (Fig. S7A,B), as well as CDH11‐CTF1 in the nucleus as evidenced by western blot (Fig. S7C,D). In addition, immunostaining for FLAG showed that CDH11 was highly co‐localized with β‐catenin at both cell junctions and in the nuclei of MCF‐7 cells (Fig. S7E) and the fraction of Ki67^+^ cells increased significantly, indicating increased proliferation, when compared with naïve MCF‐7 (Fig. S7E,F). Likewise, overexpression of CDH11 in MDA‐MB‐231 led to increased accumulation of nuclear β‐catenin and CDH11‐CTF1 when compared with naïve MDA‐MB‐231 (Fig. S8A–D).

Notably, immunoprecipitation (IP) using antibodies against FLAG or β‐catenin showed that both CDH11‐CTF1 and CDH11‐CTF2 but not CDH11‐CTF3 bound to β‐catenin not only at the cell membrane but also the cytoplasm and the nucleus in both cell types (Fig. 7E,F), suggesting that CDH11‐CTF1/2 and β‐catenin may co‐transport to the nucleus as a complex. More studies are necessary to identify the CTF binding sites where β‐catenin binds to and disrupt the binding to show that it is necessary to stabilize β‐catenin and activate the Wnt pathway.

## 4 Discussion

**Fig. 8:**
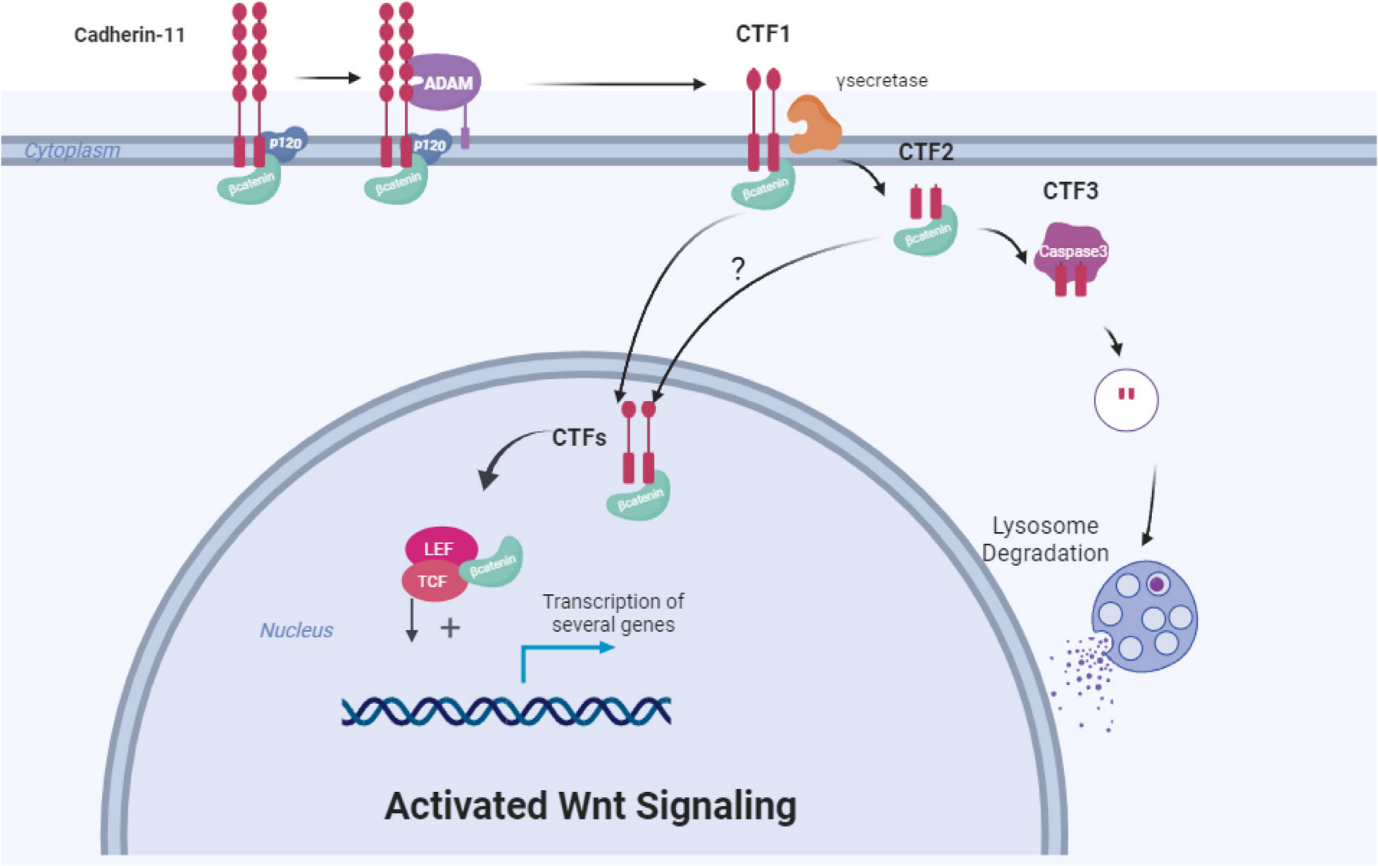
Schematic depicting the plausible mechanism of CDH11 in regulation of Wnt pathway. The intracellular domain of CDH11 or CDH11‐CTFs binds to β‐catenin, translocates to the nucleus and activates the Wnt signaling pathway, promoting proliferation, migration and invasiveness of breast cancer cells.

In this study, we examined both transcriptional and protein levels of CDH11 in multiple tumor types and reported that a high percentage of CDH11^+^ cells reside in a wide spectrum of tumors including brain, adrenal gland, skin, testis, ovary and breast. Particularly, breast cancer is one of the cancer types with high CDH11 expression. Moreover, we reported a novel finding that CDH11 is localized in the cell nucleus both *in vivo* and *in vitro*, and associates with nuclear β‐catenin. We discovered that β‐catenin remains bound to carboxy‐terminal fragments (CTF1/2) resulting from cleavage of CDH11 at the cell membrane, and the CDH11‐CTFs/β‐catenin complex serves to stabilize β‐catenin in the cytoplasm, which facilitates the subsequent translocation to the nucleus and activation of Wnt signaling (Fig. 8).

CDH11 is known as a transmembrane protein mediating cellular adhesion and a hallmark of mesenchymal phenotype that is broadly expressed in multiple cell types undergoing EMT including breast [17, 18, 20, 21], prostate [22, 23], brain [40], renal [24], bone [25], bladder [41], ovary [42] and gastric [43, 44] cancer cells. Although some studies reported that *CDH11* is frequently methylated and silenced in certain types of cancers [45, 46, 47], cumulative emerging evidence suggests that CDH11 may play a critical role in promoting tumorigenesis and metastasis in breast cancer. CDH11 has been documented to be preferentially expressed in clinical specimens of malignant when compared with non‐malignant breast tumor or normal tissue [15]. Indeed, CDH11 has been found to be expressed in most invasive breast cancer cell lines and has been proposed as a hallmark of invasiveness [17]. Increased CDH11 expression in breast cancer has been associated with poor prognosis and lower overall survival rates [16]. *In vitro*, overexpression of CDH11 in non‐metastatic breast cancer cell line MCF‐7 dramatically promotes cell migration and invasion potential [18]. Similarly, up‐regulation of CDH11 enhances cell motility and promotes metastasis of renal cell carcinoma and prostate cancer cells to bone [22, 24]. Conversely, disruption of CDH11 function by a neutralizing antibody or knockdown using siRNA significantly reduces tumorigenicity and metastasis of TNBC cells [16, 19, 25]. Previous studies from our lab demonstrated that CDH11 plays a crucial role in increasing cell proliferation via activation of the pro‐survival AKT signaling pathway by sensitizing cells to growth factor signaling [27], as well as promoting extracellular matrix (ECM) synthesis by coordinating the TGF‐β and ROCK signaling pathways [28], which may contribute to the essential role of CDH11 in facilitating tumorigenesis and metastasis. Most notably, our current results show that CDH11 co‐localizes with β‐catenin in the nucleus, while depletion of CDH11 or inhibition of CDH11 cleavage diminishes Wnt activation with a concomitant decrease in the proliferation, migration and invasion capacity of cancer cells.

Although aberrant Wnt pathway activity has been well documented in TNBC [5, 6] the potential role of CDH11 in Wnt pathway activation has not been investigated. In this study, we demonstrate that full‐length CDH11 remains on the cell surface, while CDH11‐ID or CTFs accumulate in the cytoplasm and the nucleus, where they co‐localize with β‐catenin. This result is in agreement with previous reports on CDH11 cleavage in neural crest and synovial fibroblasts [38, 39, 48], with varying numbers and sizes of CDH11‐CTFs [38], possibly due to differences in protease type/activity or CTF stability. However, this is the first study reporting co‐localization of CDH11 fragments in the nucleus with β‐catenin. Since classical cadherins share a highly conserved intracellular domain containing both P120 and β‐catenin binding domains [37], CDH11 may experience similar cleavage events as E‐ or N‐cadherin, which are sequentially cleaved by matrix metalloproteinase (MMPs), γ‐secretase, caspase‐3 and calpain [38, 39, 49], and have also been reported to localize in the nucleus of tumor cells [50, 51].

Indeed, IP experiments demonstrated that CDH11 intracellular fragments, CTF1 (49 kDa) and CTF2 (40 kDa) remained bound to β‐catenin but the smallest fragment CTF3 (34 kDa) did not. In addition, inhibition of ADAMs (batimastat) and γ‐secretase (DAPT), two proteases that were shown to generate CDH11‐CTF1 and CTF2 [38, 39] resulted in significantly reduced β‐catenin and CDH11‐ID accumulation in the nucleus with concomitant reduction of Wnt activity. We speculate that the formation of CDH11‐CTFs/β‐catenin complex may protect both proteins from degradation, as β‐catenin may shield the PEST sequence motif on CDH11‐CTFs, which is targeted by ubiquitin ligases for degradation; while binding to CTFs may stabilize β‐catenin by preventing targeting by the β‐catenin destruction complex [52]. More detailed biochemical studies are necessary to identify the proteases and cleavage sites as well as to establish which CTFs might be necessary for β‐catenin binding, stabilization, nuclear translocation and Wnt activation.

Interestingly, CTF bands were thicker and more intense in lysates that were prepared for subcellular fractionation assays, when compared with lysates harvested from cell monolayers. In preparation for fractionation assays, the cells were first detached from the surface by enzymatic treatment, which breaks cell–cell interactions, resulting in single cells. Breaking CDH11‐CDH11 junctions may promote subsequent CDH11 cleavage by other proteases such as ADAMs (batimastat) and γ‐secretase leading to CDH11‐CTFs generation. Although the physiological significance of such enzymatic treatment is not clear, it is possible that it mimics the tumor microenvironment, which abounds with high protease concentrations and activities [53, 54]. Therefore, enzymatic treatment could be recapitulating key aspects of the tumor microenvironment and its influence on CDH11 localization leading to nuclear accumulation of CDH11 as observed in clinical TMA samples.

In addition to the Wnt signaling pathway, CDH11‐CTFs/β‐catenin signaling may affect several other pathways such as the Hippo/YAP pathway [55, 56]. Such interactions may be bidirectional as nuclear β‐catenin acts as a transcription factor and induces expression of the YAP gene in colon cancer [57], while YAP has been shown to enhance nuclear β‐catenin in hepatoblastoma [58]. Moreover, other signaling molecules such as APC in the Wnt/ β‐catenin signaling pathway have been shown to negatively affect YAP in colon cancer [59], while MST1/2 deletion in the Hippo/YAP pathway was shown to activate β‐catenin in hepatocellular carcinoma [60]. Therefore, loss of homotypic engagement and cleavage of CDH11 may affect several pathways through β‐catenin and potentially other effectors, ultimately, affecting tumor growth and metastatic potential.

## 5 Conclusions

This is the first report on the role of CDH11 in binding and stabilizing β‐catenin, co‐transporting to the nucleus and promoting Wnt activation. Our discovery provides mechanistic insight into the role of CDH11 in tumorigenesis and metastasis and offers a plausible explanation of the clinical data correlating tumor grade and metastatic potential with the levels of CDH11 expression in breast cancer cells. Our findings may also be extended to other tumor cells with high CDH11 expression, but further studies are needed to verify this hypothesis. Finally, our results may provide novel ways to reduce tumor growth and metastatic potential by inhibiting CDH11 proteolytic degradation and subsequent translocation of β‐catenin to the nucleus.

## Conflict of interest

The authors declare no conflict of interest.

## Author contributions

YL contributed to experimental design, primary data collection, statistical analysis, as well as preparation and editing of the manuscript. PL participated in design and implementation of molecular cloning, primary data collection, wound healing experiments as well as preparation and editing of the manuscript. RZS contributed in CRISPR cell line generation and 2D/3D invasive assay. AMK participated in primary data collection and 2D/3D invasive assay. TG contributed to TMA data analysis. WB contributed to TMA preparation and data analysis. SN contributed to experimental design and manuscript editing. STA oversaw the entire study and contributed to experimental design, data interpretation, and writing and editing of the manuscript. All authors agree to the authorship as listed in the manuscript.

### Peer review

The peer review history for this article is available at https://www.webofscience.com/api/gateway/wos/peer‐review/10.1002/1878‐0261.13507.

## Supporting information


**Fig. S1.** Schematic of Tissue MicroArray (TMA) analysis.
**Fig. S2.** CDH11 expression in Clinical Samples.
**Fig. S3.** Single cell confocal images.
**Fig. S4.** Immunostaining of CDH11‐ID in MDA‐MB‐231.
**Fig. S5.** Western Blot for CDH11 in Triple‐Negative Breast Cancer cell line.
**Fig. S6.** Viability assay with additional Batmastat and DAPT.
**Fig. S7.** Detecting CDH11 fragments in MCF‐7 with overexpression CDH11.
**Fig. S8.** Detecting CDH11 fragments in MDA‐MB‐231 with overexpression CDH11.
**Table S1.** Antibody list.
**Table S2.** Forward and reverse primers for RT‐PCR of the indicated genes.

## Data Availability

The data that support the findings of this study are available from the corresponding author [sandread@buffalo.edu] upon reasonable request.
