# Supplementary material for: Cadherin‐11 increases tumor cell proliferation and metastatic potential via Wnt pathway activation
Source: Mol Oncol. 2023 Sep 8;17(10):2056–73. doi: 10.1002/1878-0261.13507 (PMC10552893; doi:10.1002/1878-0261.13507)
Supplement: Supplementary file 1 — Fig. S1. Schematic of Tissue MicroArray (TMA) analysis. Fig. S2. CDH11 expression in Clinical Samples. Fig. S3. Single cell confocal images. Fig. S4. Immunostaining of CDH11‐ID in MDA‐MB‐231. Fig. S5. Western Blot for CDH11 in Triple‐Negative Breast Cancer cell line. Fig. S6. Viability assay with additional Batmastat and DAPT. Fig. S7. Detecting CDH11 fragments in MCF‐7 with overexpression CDH11. Fig. S8. Detecting CDH11 fragments in MDA‐MB‐231 with overexpression CDH11. Table S1. Antibody list. Table S2. Forward and reverse primers for RT‐PCR of the indicated genes. [file MOL2-17-2056-s001.pptx]

## Slide 1
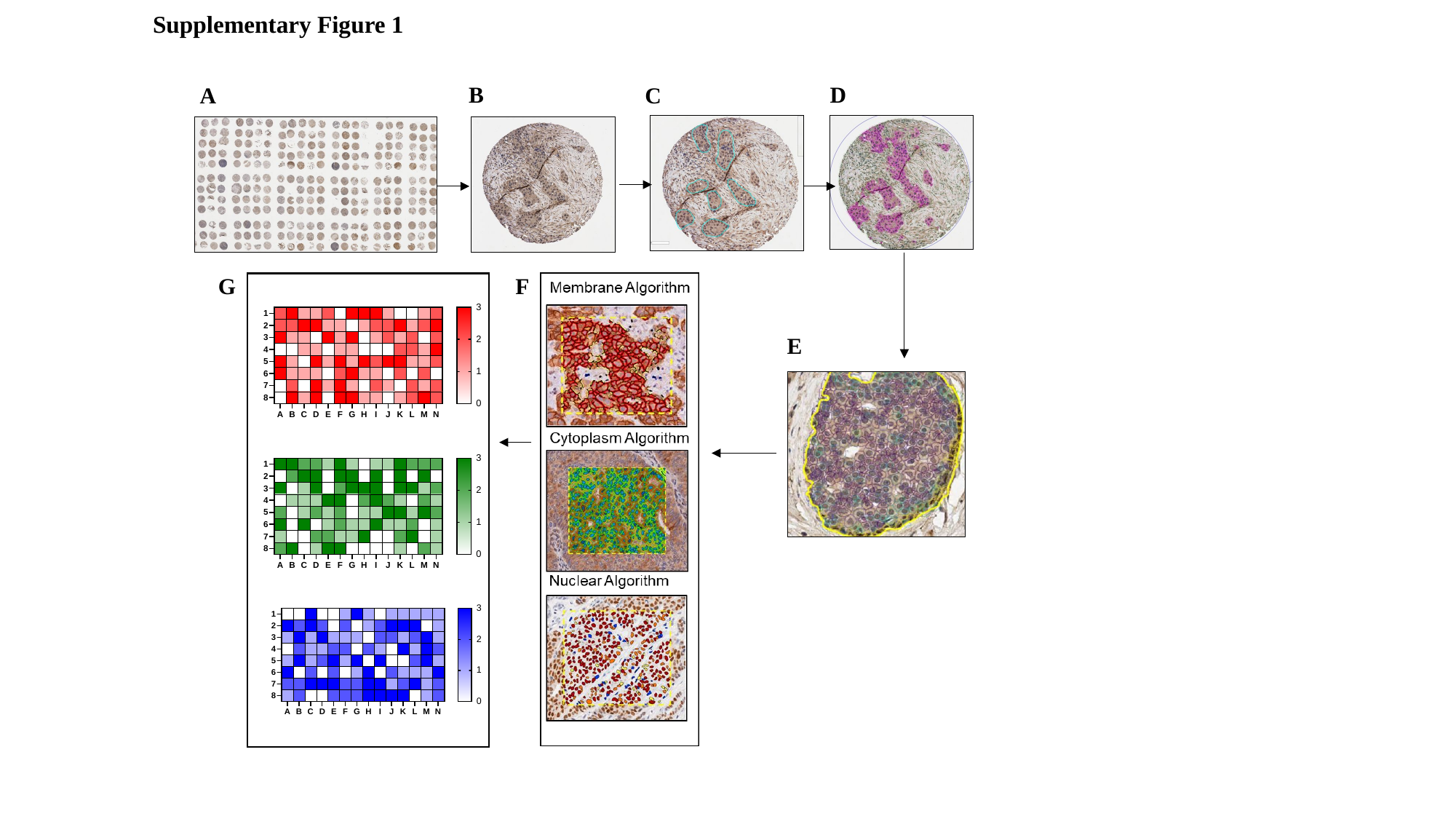

Supplementary Figure 1
B
D
C
A
G
F
E

## Slide 2
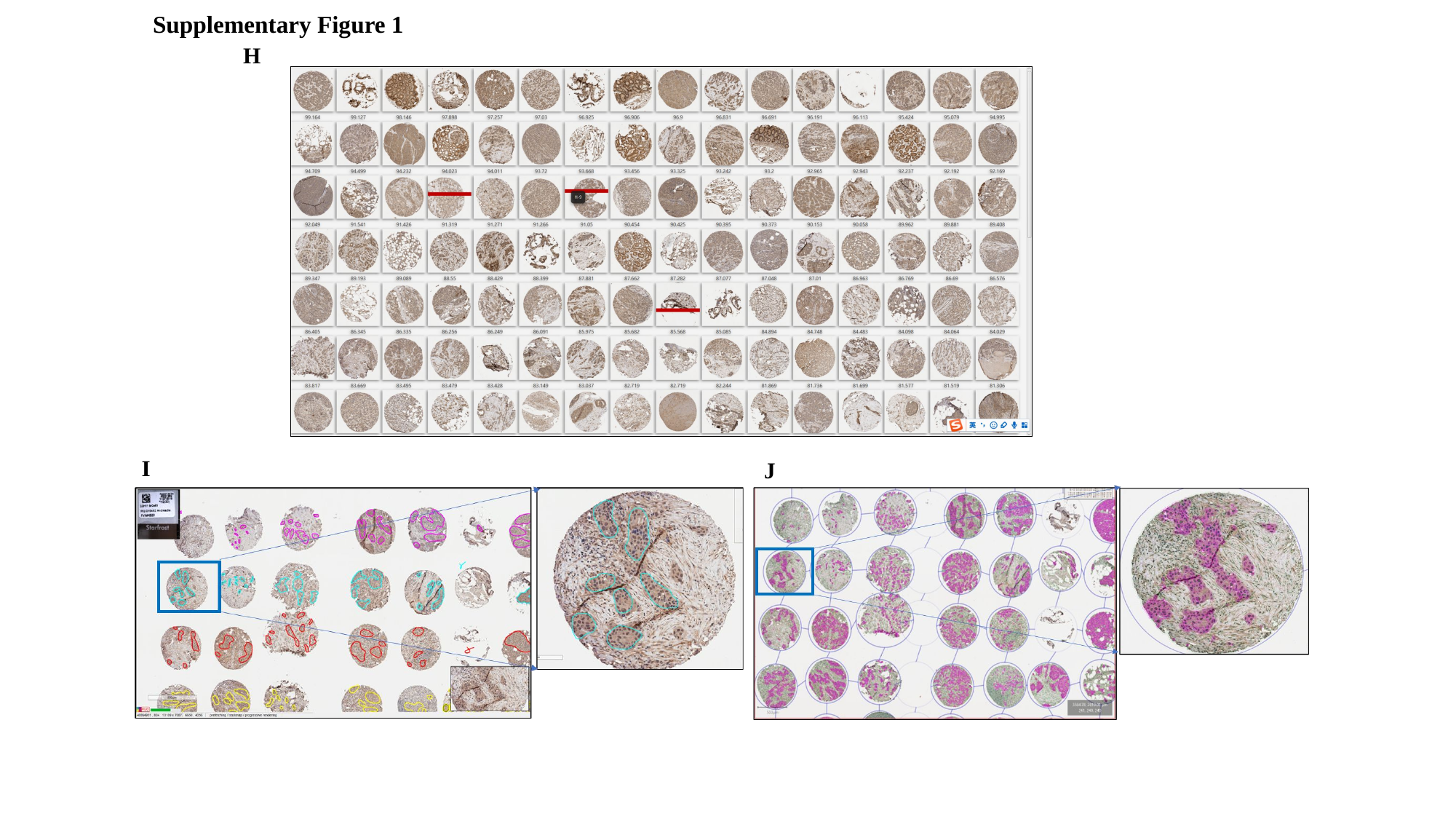

Supplementary Figure 1
H
I
J

## Slide 3
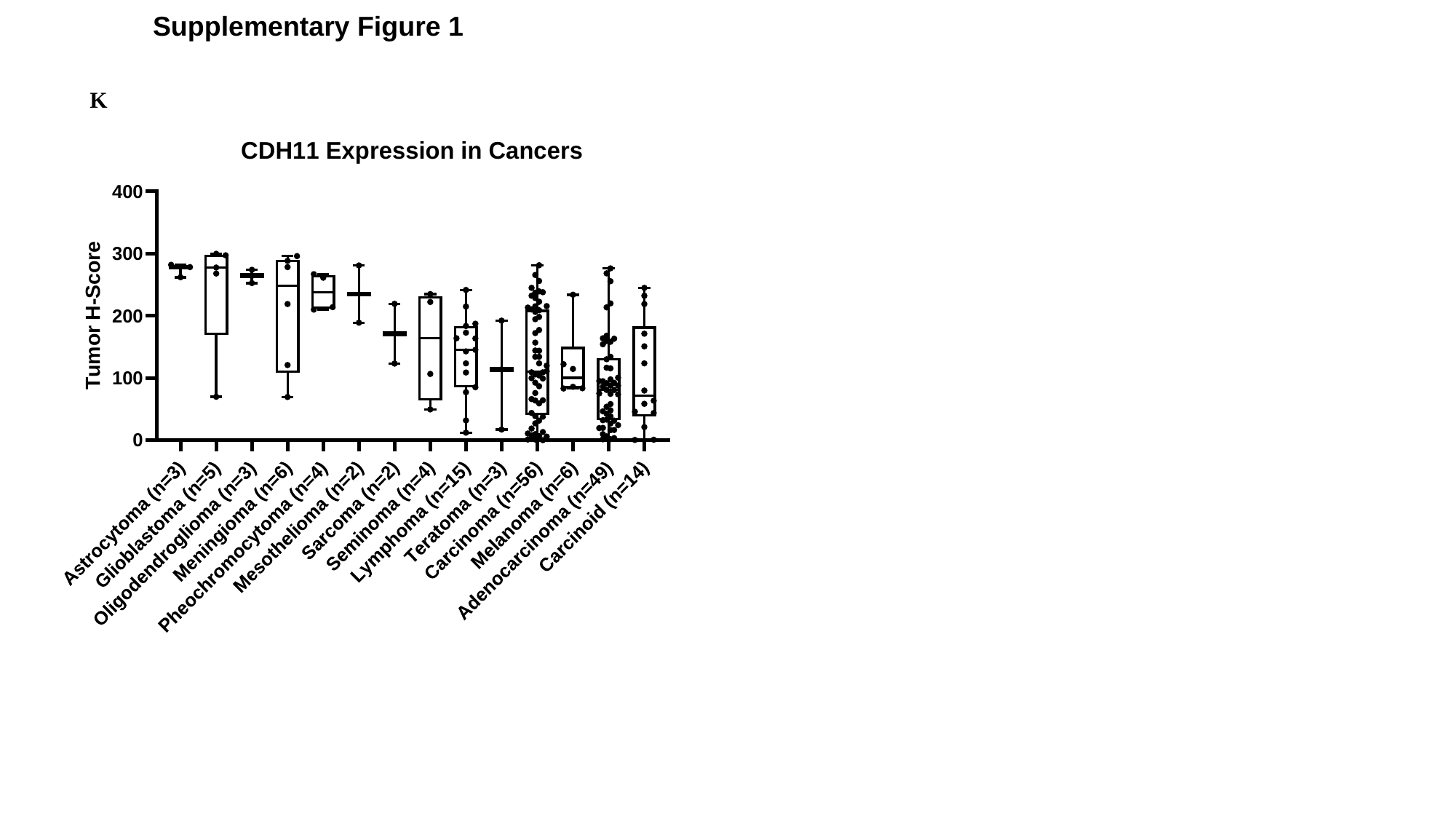

Supplementary Figure 1
K

## Slide 4
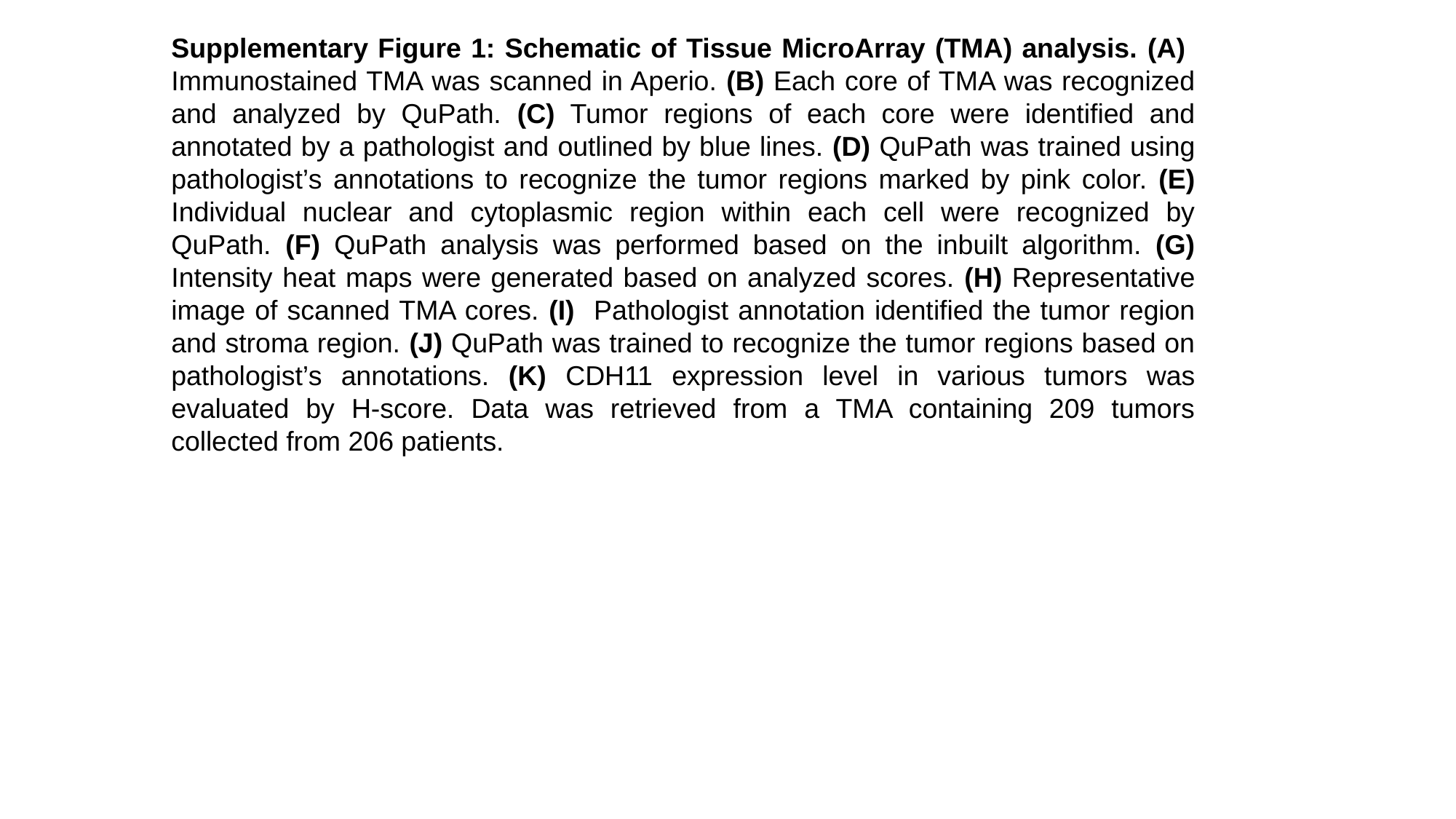

Supplementary Figure 1: Schematic of Tissue MicroArray (TMA) analysis. (A) Immunostained TMA was scanned in Aperio. (B) Each core of TMA was recognized and analyzed by QuPath. (C) Tumor regions of each core were identified and annotated by a pathologist and outlined by blue lines. (D) QuPath was trained using pathologist’s annotations to recognize the tumor regions marked by pink color. (E) Individual nuclear and cytoplasmic region within each cell were recognized by QuPath. (F) QuPath analysis was performed based on the inbuilt algorithm. (G) Intensity heat maps were generated based on analyzed scores. (H) Representative image of scanned TMA cores. (I) Pathologist annotation identified the tumor region and stroma region. (J) QuPath was trained to recognize the tumor regions based on pathologist’s annotations. (K) CDH11 expression level in various tumors was evaluated by H-score. Data was retrieved from a TMA containing 209 tumors collected from 206 patients.

## Slide 5
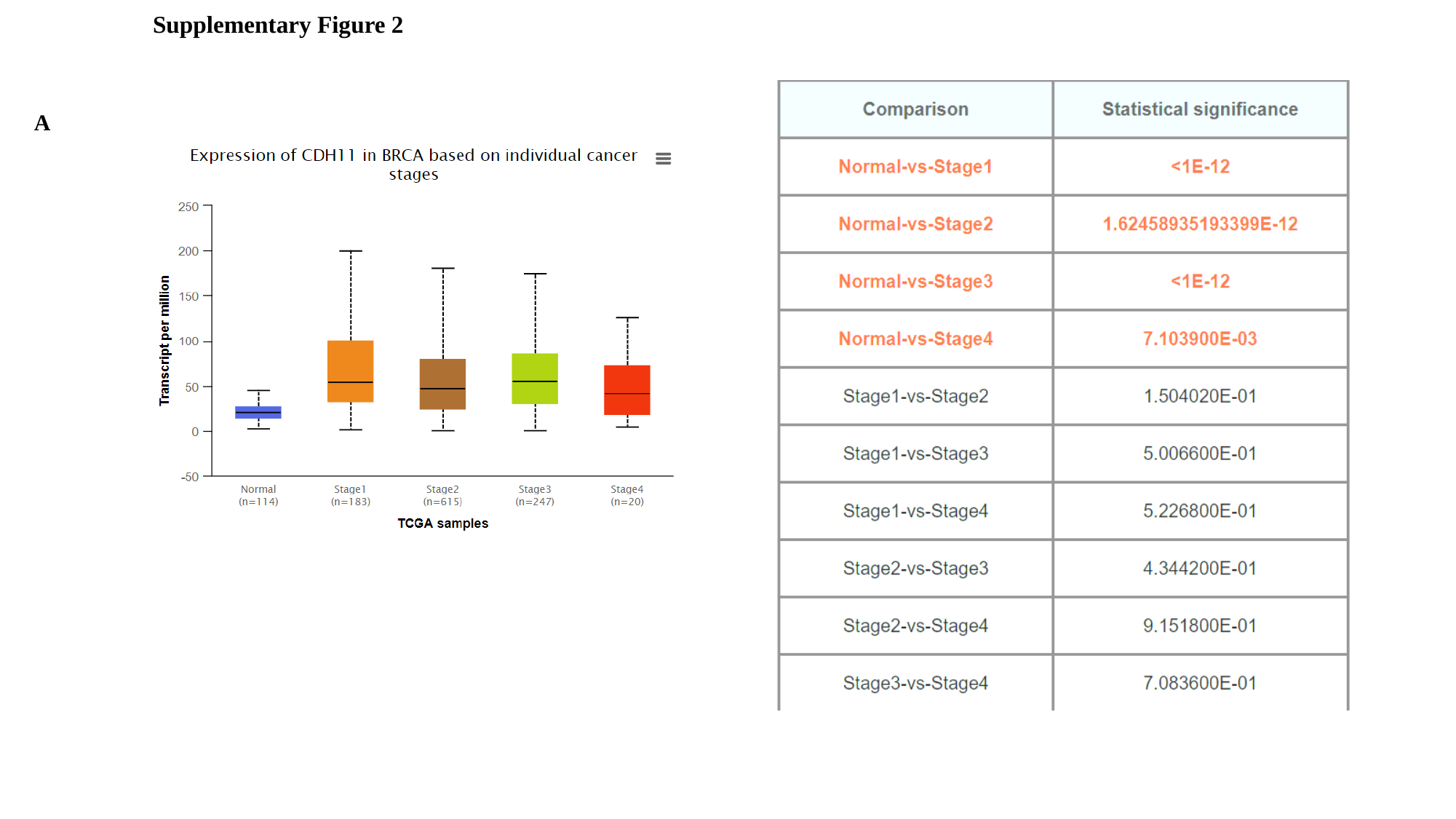

Supplementary Figure 2
A

## Slide 6
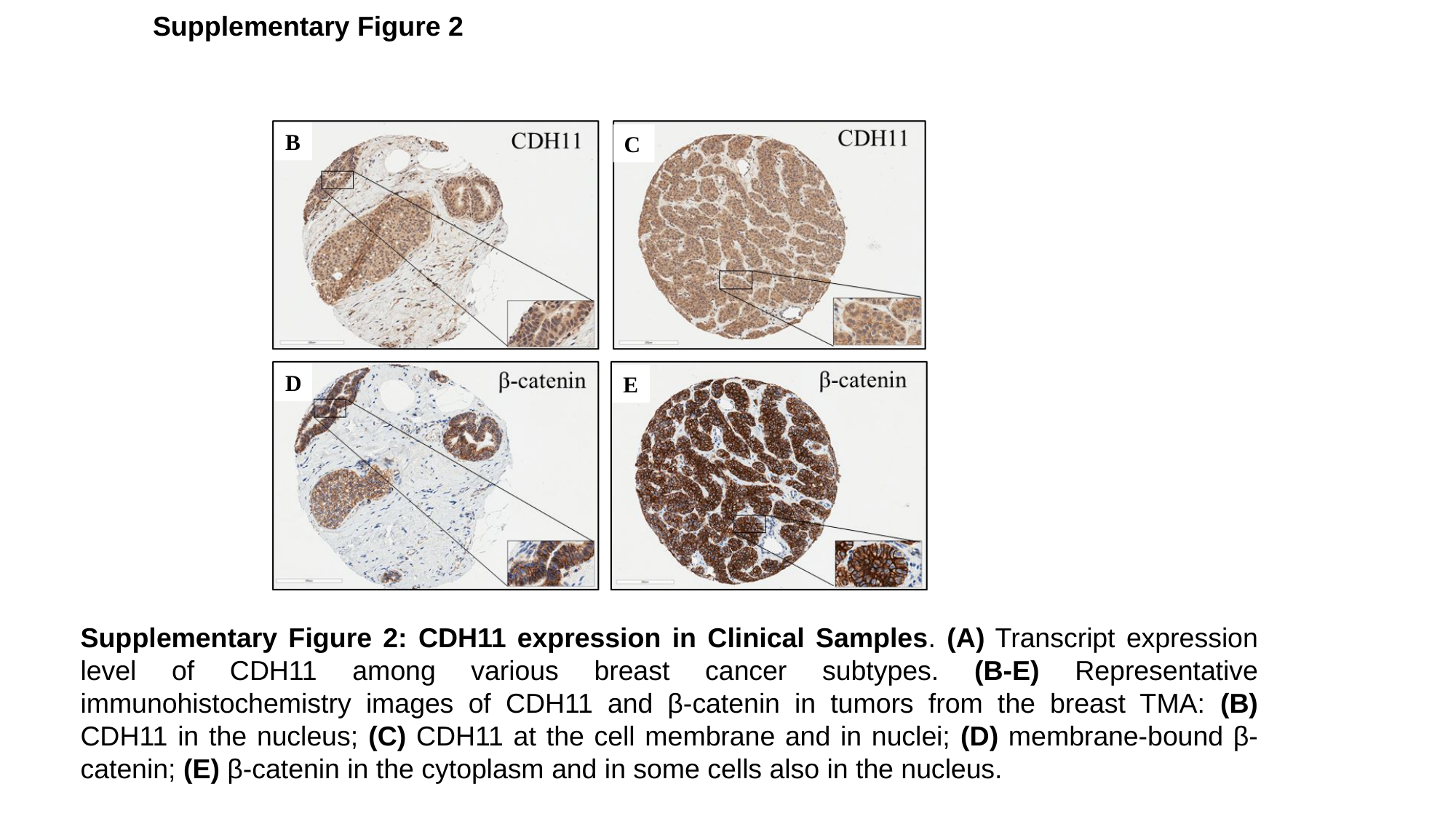

Supplementary Figure 2
B
C
D
E
Supplementary Figure 2: CDH11 expression in Clinical Samples. (A) Transcript expression level of CDH11 among various breast cancer subtypes. (B-E) Representative immunohistochemistry images of CDH11 and β-catenin in tumors from the breast TMA: (B) CDH11 in the nucleus; (C) CDH11 at the cell membrane and in nuclei; (D) membrane-bound β-catenin; (E) β-catenin in the cytoplasm and in some cells also in the nucleus.

## Slide 7
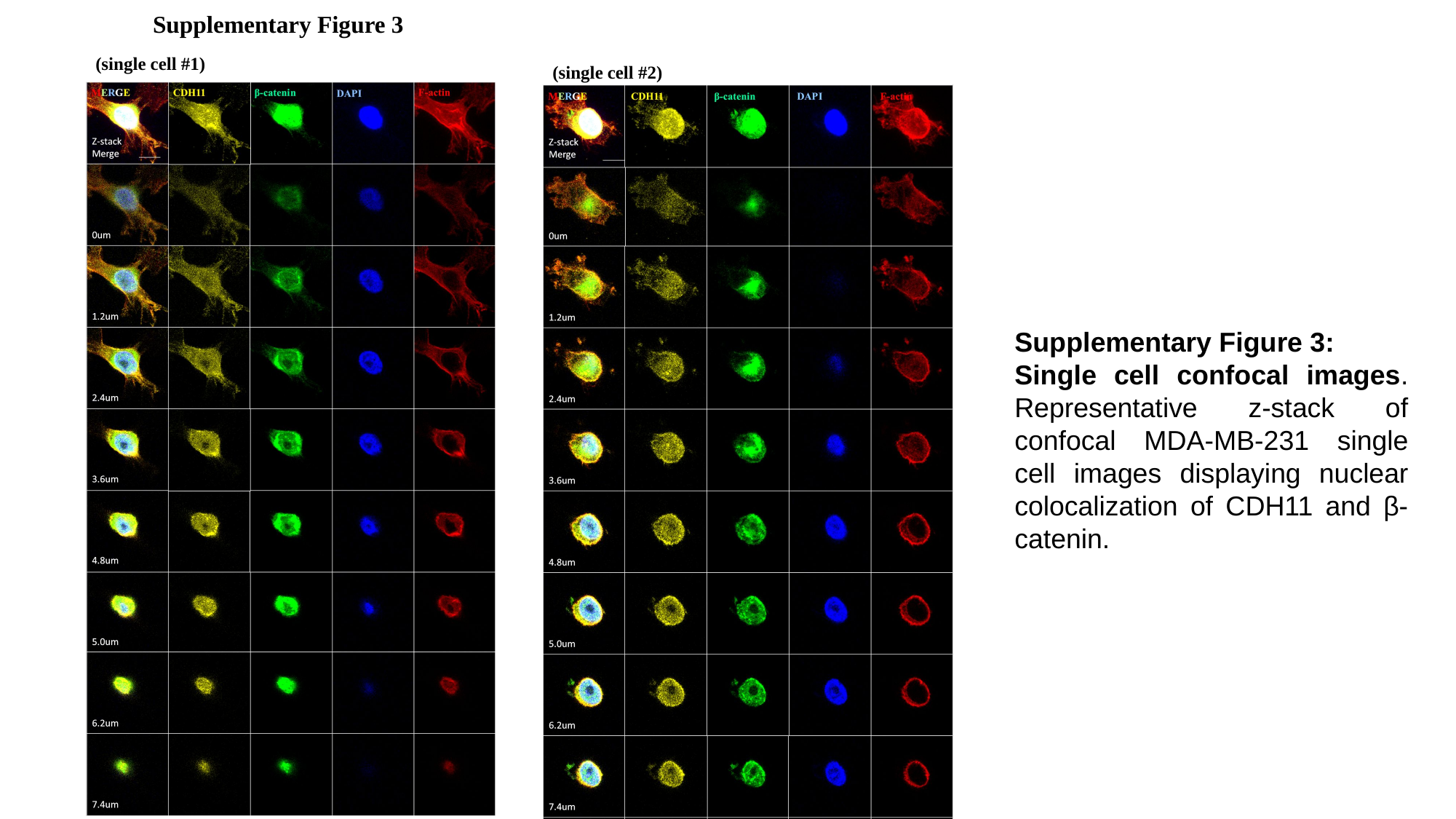

Supplementary Figure 3
(single cell #1)
(single cell #2)
Supplementary Figure 3:
Single cell confocal images. Representative z-stack of confocal MDA-MB-231 single cell images displaying nuclear colocalization of CDH11 and β-catenin.

## Slide 8
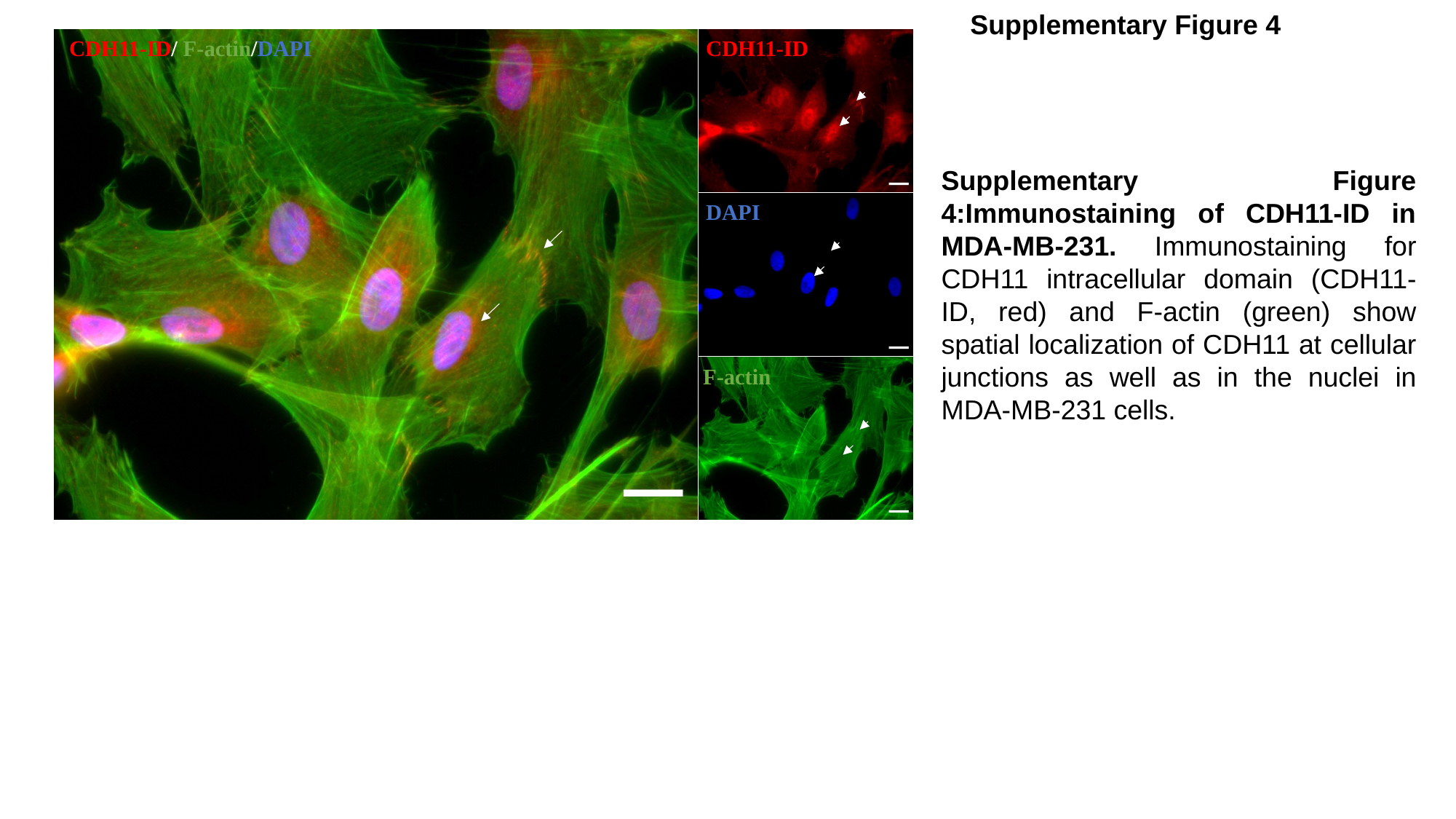

Supplementary Figure 4
CDH11-ID/ F-actin/DAPI
CDH11-ID
Supplementary Figure 4:Immunostaining of CDH11-ID in MDA-MB-231. Immunostaining for CDH11 intracellular domain (CDH11-ID, red) and F-actin (green) show spatial localization of CDH11 at cellular junctions as well as in the nuclei in MDA-MB-231 cells.
DAPI
F-actin

## Slide 9
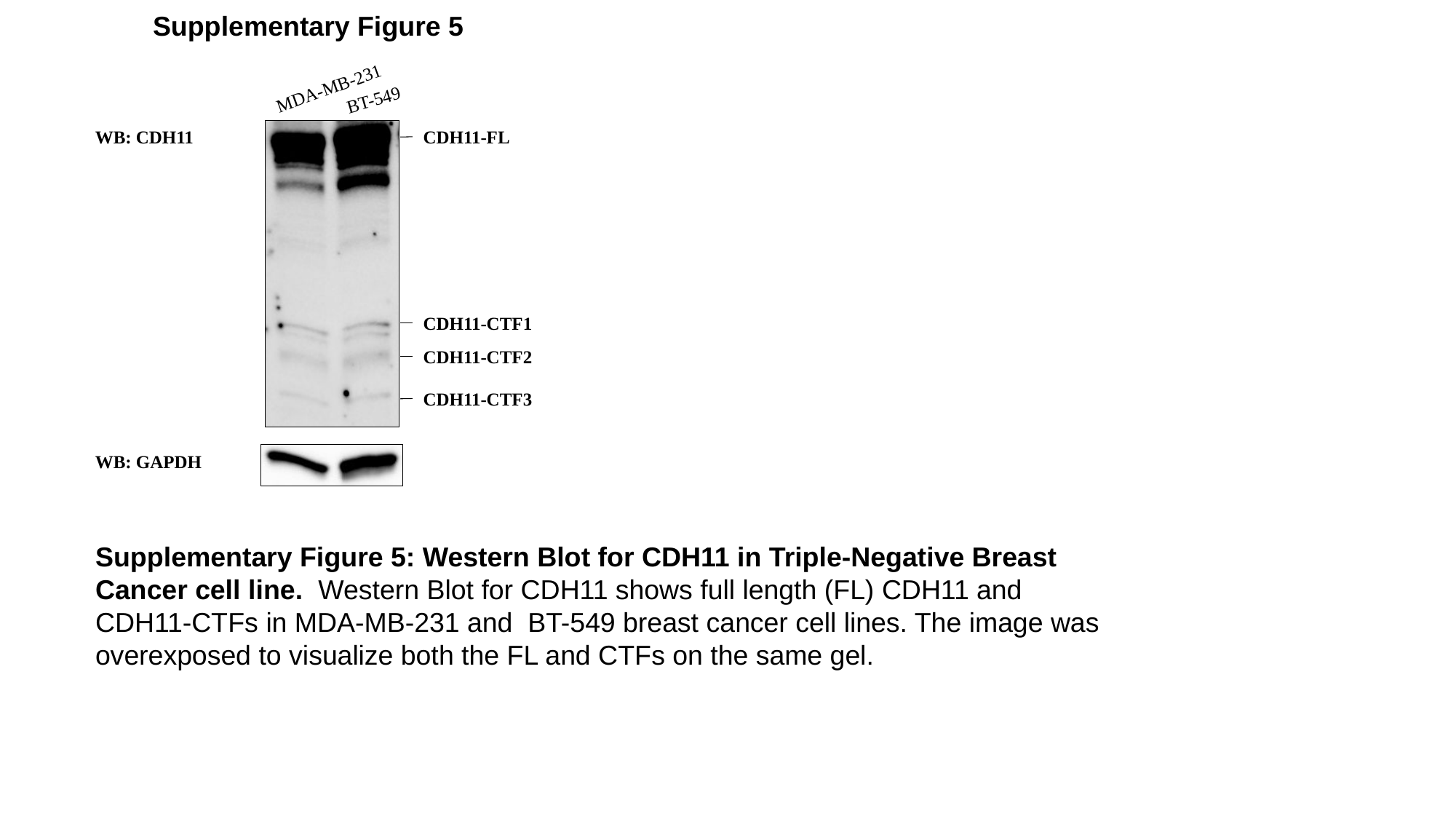

Supplementary Figure 5
MDA-MB-231
BT-549
WB: CDH11
CDH11-FL
CDH11-CTF1
CDH11-CTF2
CDH11-CTF3
WB: GAPDH
Supplementary Figure 5: Western Blot for CDH11 in Triple-Negative Breast Cancer cell line. Western Blot for CDH11 shows full length (FL) CDH11 and CDH11-CTFs in MDA-MB-231 and BT-549 breast cancer cell lines. The image was overexposed to visualize both the FL and CTFs on the same gel.

## Slide 10
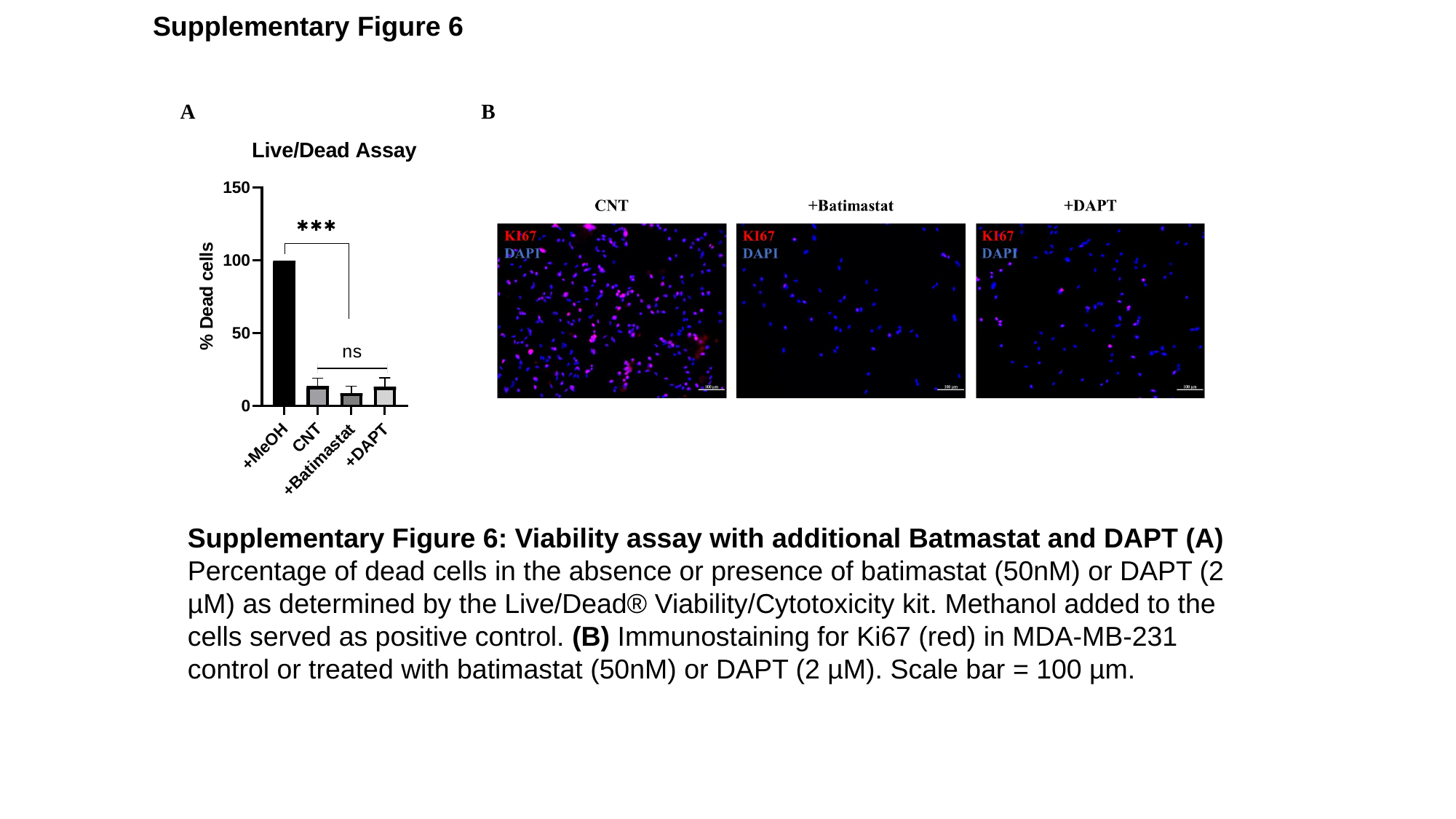

Supplementary Figure 6
A
B
Supplementary Figure 6: Viability assay with additional Batmastat and DAPT (A) Percentage of dead cells in the absence or presence of batimastat (50nM) or DAPT (2 µM) as determined by the Live/Dead® Viability/Cytotoxicity kit. Methanol added to the cells served as positive control. (B) Immunostaining for Ki67 (red) in MDA-MB-231 control or treated with batimastat (50nM) or DAPT (2 µM). Scale bar = 100 µm.

## Slide 11
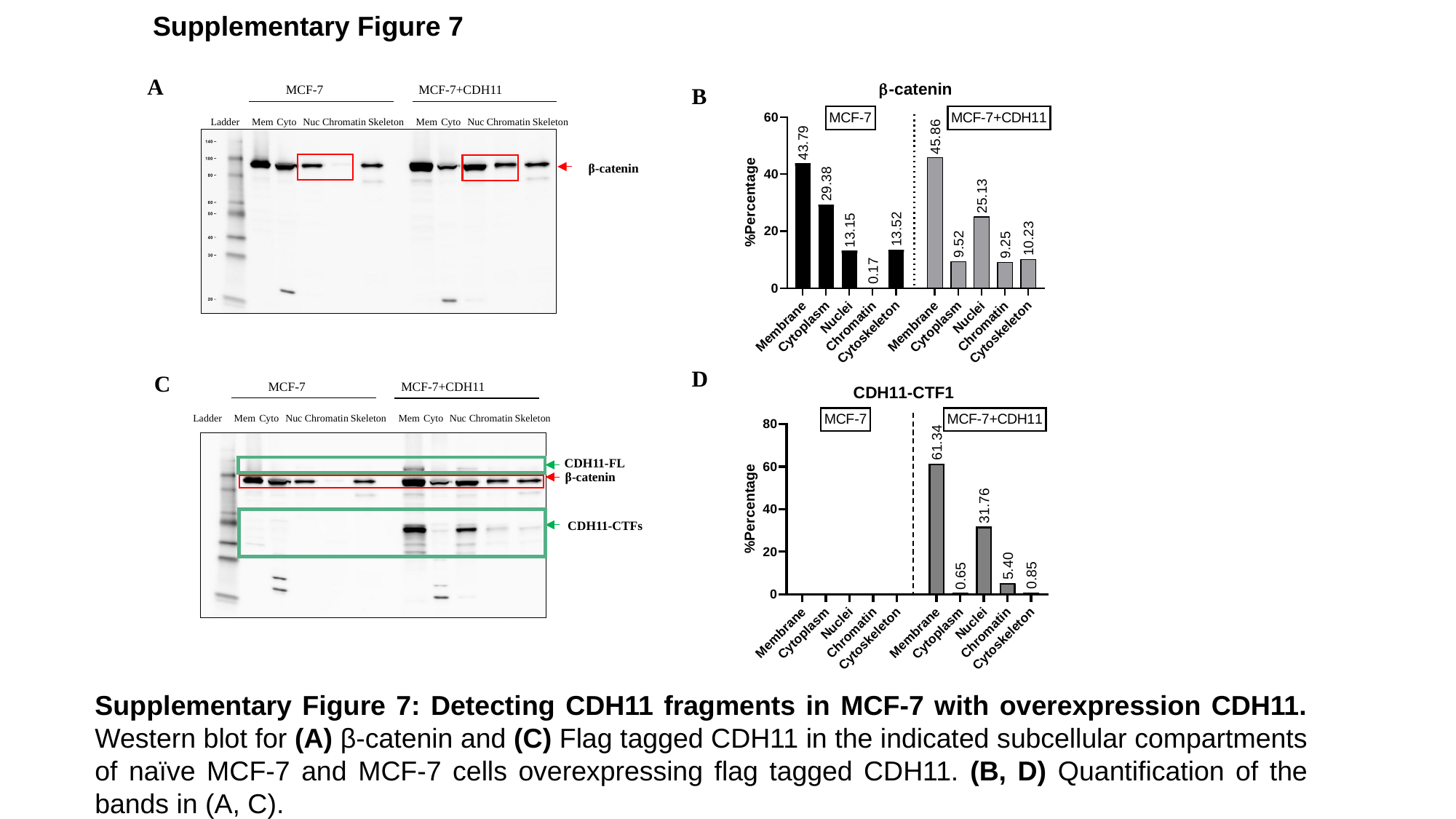

Supplementary Figure 7
A
MCF-7
MCF-7+CDH11
B
Chromatin
Skeleton
Chromatin
Skeleton
Ladder
Mem
Cyto
Nuc
Mem
Cyto
Nuc
β-catenin
D
C
MCF-7
MCF-7+CDH11
Chromatin
Skeleton
Chromatin
Skeleton
Ladder
Mem
Cyto
Nuc
Mem
Cyto
Nuc
CDH11-FL
β-catenin
 CDH11-CTFs
Supplementary Figure 7: Detecting CDH11 fragments in MCF-7 with overexpression CDH11. Western blot for (A) β-catenin and (C) Flag tagged CDH11 in the indicated subcellular compartments of naïve MCF-7 and MCF-7 cells overexpressing flag tagged CDH11. (B, D) Quantification of the bands in (A, C).

## Slide 12
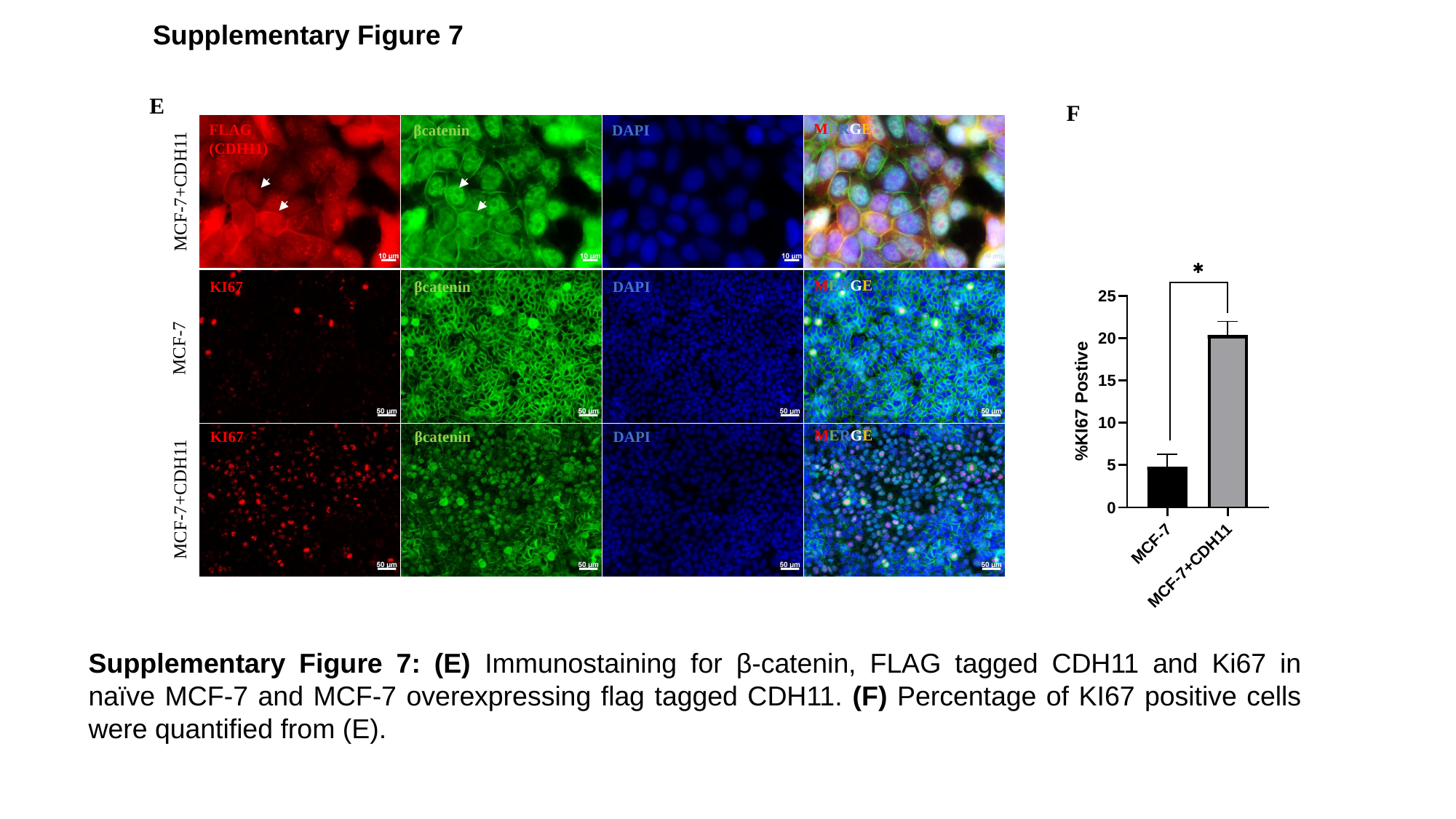

Supplementary Figure 7
E
F
MERGE
FLAG (CDH11)
βcatenin
DAPI
MCF-7+CDH11
MERGE
KI67
βcatenin
DAPI
MCF-7
MERGE
KI67
βcatenin
DAPI
MCF-7+CDH11
Supplementary Figure 7: (E) Immunostaining for β-catenin, FLAG tagged CDH11 and Ki67 in naïve MCF-7 and MCF-7 overexpressing flag tagged CDH11. (F) Percentage of KI67 positive cells were quantified from (E).

## Slide 13
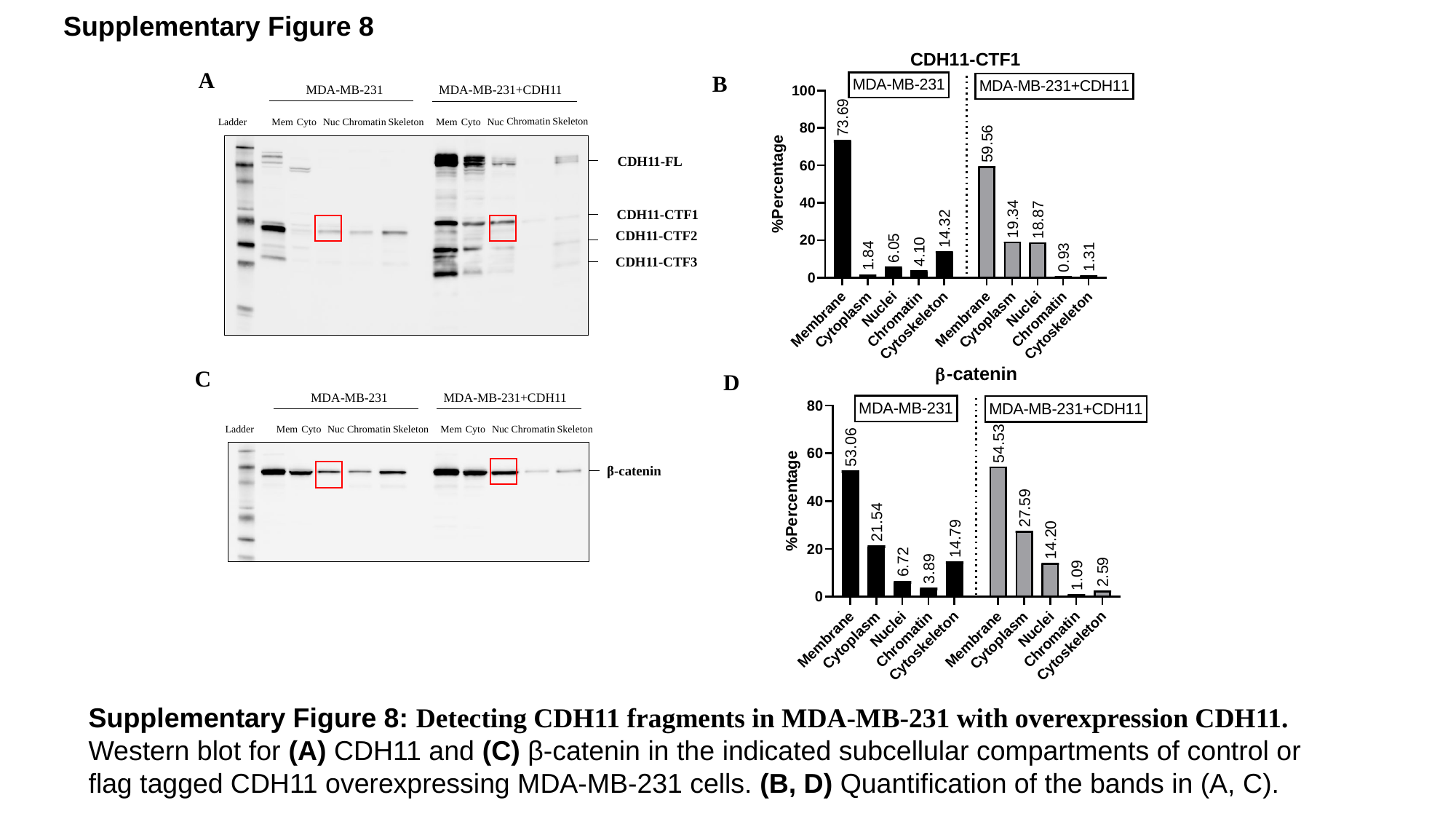

Supplementary Figure 8
A
B
MDA-MB-231
MDA-MB-231+CDH11
Chromatin
Skeleton
Chromatin
Skeleton
Mem
Cyto
Nuc
Ladder
Mem
Cyto
Nuc
CDH11-FL
CDH11-CTF1
CDH11-CTF2
CDH11-CTF3
C
D
MDA-MB-231
MDA-MB-231+CDH11
Chromatin
Skeleton
Chromatin
Skeleton
Mem
Cyto
Nuc
Ladder
Mem
Cyto
Nuc
β-catenin
Supplementary Figure 8: Detecting CDH11 fragments in MDA-MB-231 with overexpression CDH11.
Western blot for (A) CDH11 and (C) β-catenin in the indicated subcellular compartments of control or flag tagged CDH11 overexpressing MDA-MB-231 cells. (B, D) Quantification of the bands in (A, C).

## Slide 14
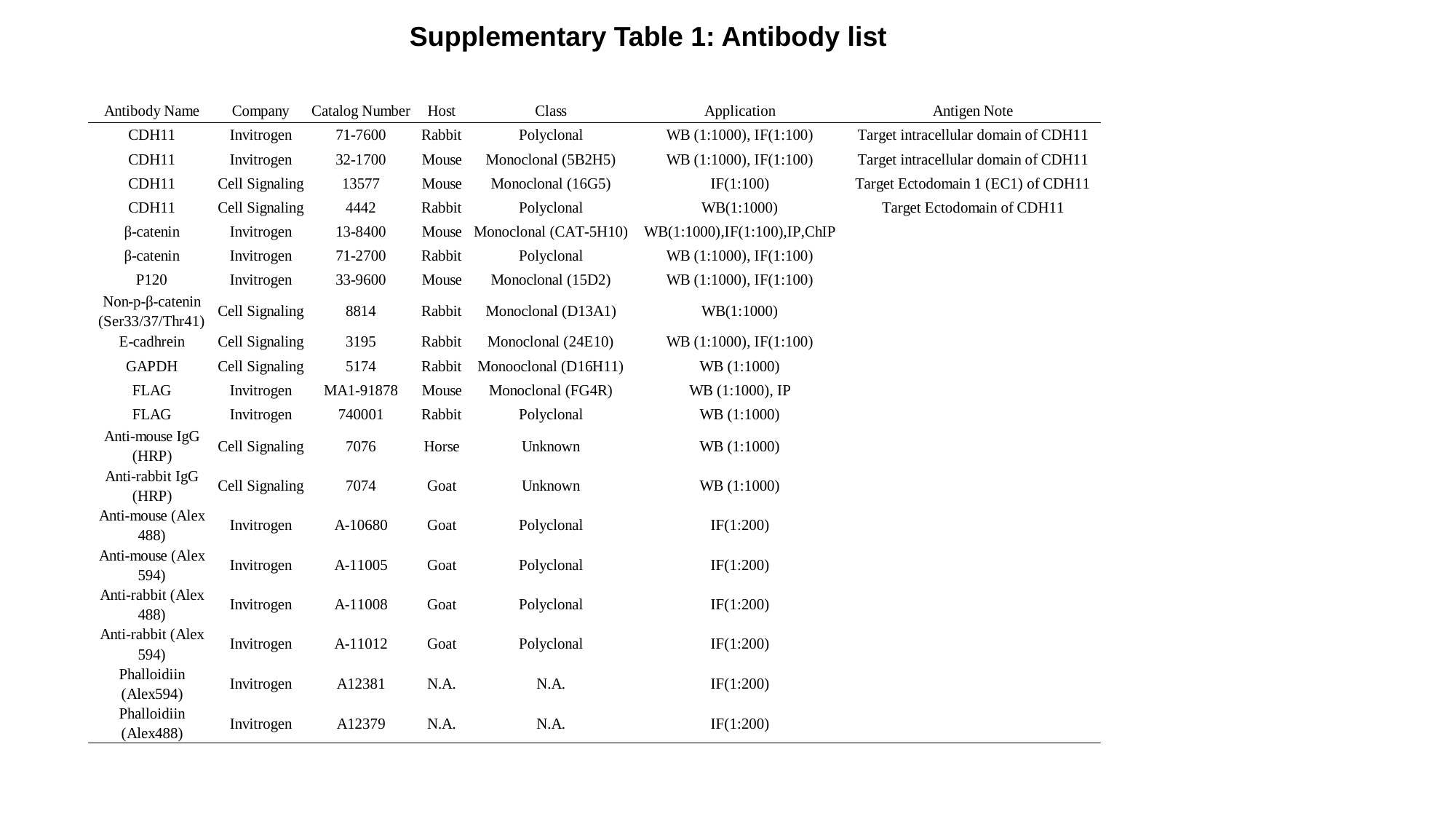

Supplementary Table 1: Antibody list

## Slide 15
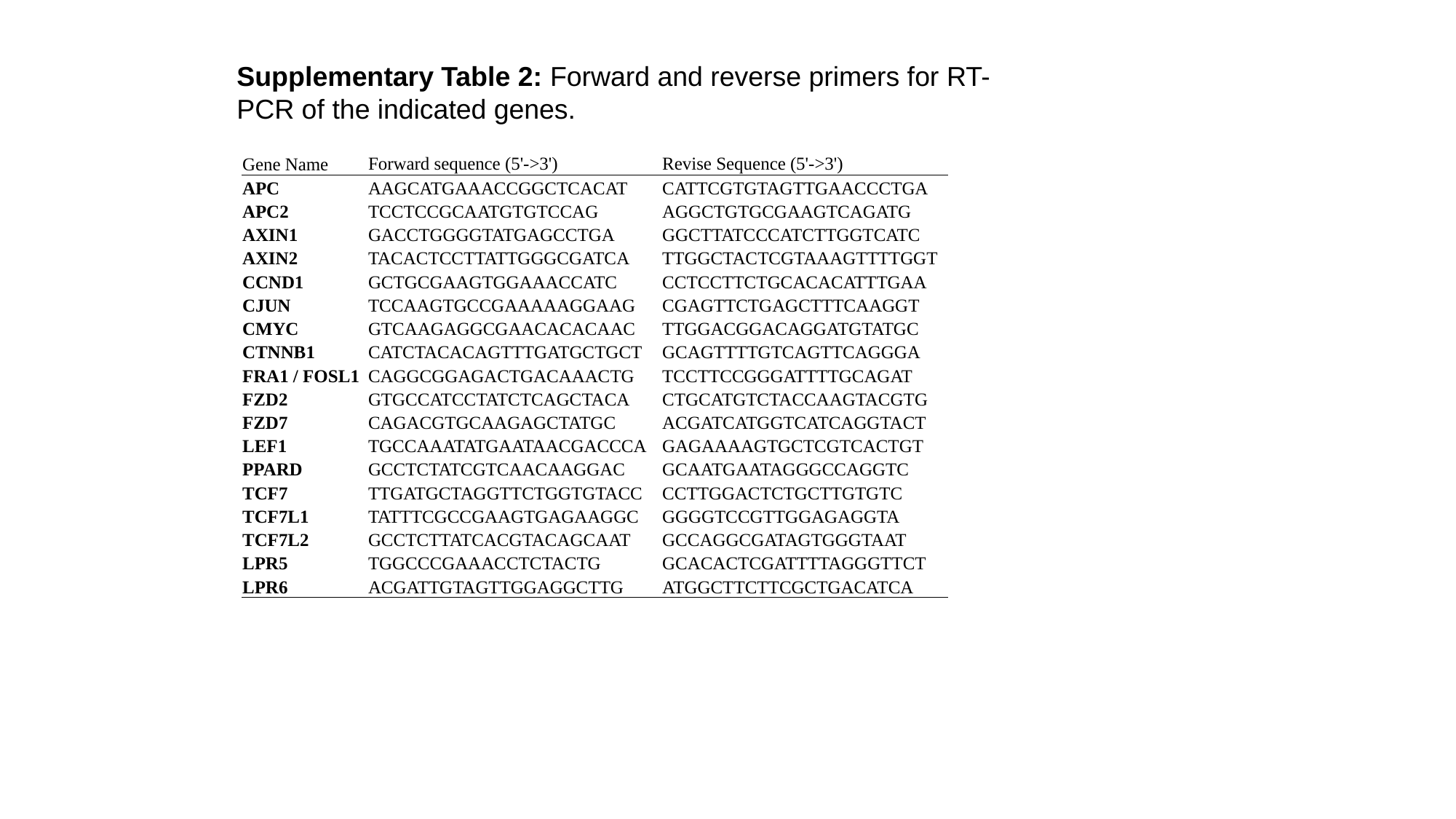

Supplementary Table 2: Forward and reverse primers for RT-PCR of the indicated genes.
| Gene Name | Forward sequence (5'->3') | Revise Sequence (5'->3') |
| --- | --- | --- |
| APC | AAGCATGAAACCGGCTCACAT | CATTCGTGTAGTTGAACCCTGA |
| APC2 | TCCTCCGCAATGTGTCCAG | AGGCTGTGCGAAGTCAGATG |
| AXIN1 | GACCTGGGGTATGAGCCTGA | GGCTTATCCCATCTTGGTCATC |
| AXIN2 | TACACTCCTTATTGGGCGATCA | TTGGCTACTCGTAAAGTTTTGGT |
| CCND1 | GCTGCGAAGTGGAAACCATC | CCTCCTTCTGCACACATTTGAA |
| CJUN | TCCAAGTGCCGAAAAAGGAAG | CGAGTTCTGAGCTTTCAAGGT |
| CMYC | GTCAAGAGGCGAACACACAAC | TTGGACGGACAGGATGTATGC |
| CTNNB1 | CATCTACACAGTTTGATGCTGCT | GCAGTTTTGTCAGTTCAGGGA |
| FRA1 / FOSL1 | CAGGCGGAGACTGACAAACTG | TCCTTCCGGGATTTTGCAGAT |
| FZD2 | GTGCCATCCTATCTCAGCTACA | CTGCATGTCTACCAAGTACGTG |
| FZD7 | CAGACGTGCAAGAGCTATGC | ACGATCATGGTCATCAGGTACT |
| LEF1 | TGCCAAATATGAATAACGACCCA | GAGAAAAGTGCTCGTCACTGT |
| PPARD | GCCTCTATCGTCAACAAGGAC | GCAATGAATAGGGCCAGGTC |
| TCF7 | TTGATGCTAGGTTCTGGTGTACC | CCTTGGACTCTGCTTGTGTC |
| TCF7L1 | TATTTCGCCGAAGTGAGAAGGC | GGGGTCCGTTGGAGAGGTA |
| TCF7L2 | GCCTCTTATCACGTACAGCAAT | GCCAGGCGATAGTGGGTAAT |
| LPR5 | TGGCCCGAAACCTCTACTG | GCACACTCGATTTTAGGGTTCT |
| LPR6 | ACGATTGTAGTTGGAGGCTTG | ATGGCTTCTTCGCTGACATCA |
